# Trends Over Time in the Prevalence of Autism by Adaptive and Intellectual Functioning Levels

**DOI:** 10.1002/aur.70167

**Published:** 2025-12-28

**Authors:** Sarah M. Furnier, Ronald Gangnon, Maureen S. Durkin

**Affiliations:** ^1^ Department of Population Health Sciences University of Wisconsin‐Madison Madison Wisconsin USA; ^2^ Waisman Center University of Wisconsin‐Madison Madison Wisconsin USA; ^3^ Department of Biostatistics and Medical Informatics University of Wisconsin‐Madison Madison Wisconsin USA

**Keywords:** adaptive behavior, adaptive functioning, co‐occurring conditions, intellectual disability, IQ, prevalence

## Abstract

The autistic community is a large, growing, and heterogeneous population, and there is a need for improved methods to describe their diverse needs. Measures of adaptive functioning collected through public health surveillance may provide valuable information on functioning and support needs at a population level. We aimed to use adaptive behavior and cognitive scores abstracted from health and educational records to describe trends over time in the population prevalence of autism by adaptive level and co‐occurrence of intellectual disability (ID). Using data from the Autism and Developmental Disabilities Monitoring Network, years 2000 to 2016, we estimated the prevalence of autism per 1000 8‐year‐old children by four levels of adaptive challenges (moderate to profound, mild, borderline, or none) and by co‐occurrence of ID. The prevalence of autism with mild, borderline, or no significant adaptive challenges increased between 2000 and 2016, from 5.1 per 1000 (95% confidence interval [CI]: 4.6–5.5) to 17.6 (95% CI: 17.1–18.1) while the prevalence of autism with moderate to profound challenges decreased slightly, from 1.5 (95% CI: 1.2–1.7) to 1.2 (95% CI: 1.1–1.4). The prevalence increase was greater for autism without co‐occurring ID than for autism with co‐occurring ID. The increase in autism prevalence between 2000 and 2016 was confined to autism with milder phenotypes. This trend could indicate improved identification of milder forms of autism over time. It is possible that increased access to therapies that improve intellectual and adaptive functioning of children diagnosed with autism also contributed to the trends.

## Lay Summary

Autism has become more common over time, especially since the early 1990s. Between 2000 and 2016, the frequency of autism among 8 year‐old children in the United States nearly tripled. During the same time period, the proportion of autistic children with significantly below average intellectual functioning decreased. We found that all of the increase in autism frequency between 2000 and 2016 was for autism with mild or no significant adaptive challenges. Also, the frequency of autism with the highest level of adaptive challenges declined slightly during this time. These findings may be due to improved recognition over time of autistic children with few adaptive challenges. They could also indicate improvements over time in the functioning of autistic children due to better access to effective therapies.

## 1 Introduction

In its fifth edition, the Diagnostic and Statistical Manual of Mental Disorders (DSM‐5) (American Psychiatric Association 2013) combined the diagnoses of Asperger's syndrome, autistic disorder, and pervasive developmental disorder into one—autism spectrum disorder (ASD). Subsequently, children diagnosed with ASD comprise a large and highly heterogeneous group with diverse strengths and challenges as well as varying types and levels of support needed. As the prevalence of autism has increased over the last several decades, in the United States (US) and globally, and as identification strategies have improved, a challenge in autism policy and epidemiology is to accurately and adequately describe the needs of the growing population of autistic children. The DSM‐5 describes autism severity levels in terms of level of support required, ranging from “requiring support” to “requiring very substantial support.” (American Psychiatric Association 2013) On a population level, these severity levels are rarely, if ever, available. Since 2000, the Centers for Disease Control and Prevention's (CDC) Autism and Developmental Disabilities Monitoring Network (ADDM) has reported the percentage of autistic children in the US with co‐occurring intellectual disability (ID) based on a score ≤ 70 on their most recent intelligence test (Autism and Developmental Disabilities Monitoring Network Surveillance Year 2000 Principal Investigators, Centers for Disease Control and Prevention 2007; Autism and Developmental Disabilities Monitoring Network Surveillance Year 2002 Principal Investigators, Centers for Disease Control and Prevention 2007; Autism and Developmental Disabilities Monitoring Network Surveillance Year 2004 Principal Investigators, Centers for Disease Control and Prevention 2009; Autism and Developmental Disabilities Monitoring Network Surveillance Year 2006 Principal Investigators, Centers for Disease Control and Prevention 2009; Autism and Developmental Disabilities Monitoring Network Surveillance Year 2008 Principal Investigators, Centers for Disease Control and Prevention 2012; Developmental Disabilities Monitoring Network Surveillance Year 2010 Principal Investigators, Centers for Disease Control and Prevention 2014; Christensen et al. 2018; Baio et al. 2018; Maenner et al. 2020). Lord and colleagues proposed the term “profound autism” to identify autistic children who require 24‐h support (Lord et al. 2022), and a recent study sought to estimate the population prevalence of “profound autism” among US children defined as meeting criteria for ASD and having very low intelligence quotient (IQ; ≤ 50) and limited verbal skills using ADDM data. Similarly, in the research literature, autism severity is often described in terms of the co‐occurrence of ID (Maenner et al. 2023; Fombonne 2003; Zeidan et al. 2022) or labels like “high‐” or “low‐functioning,” (Griesi‐Oliveira et al. 2021; Del Valle Rubido et al. 2020; Kimura et al. 2019; Schaller et al. 2019) designations often based solely on IQ scores. Autism symptom severity levels can also be derived from the Autism Diagnostic Observation Schedule (ADOS) when this instrument is used for assessment (Gotham et al. 2009). However, recent debates surrounding resource allocation for autism research, education, and support (Smith 2024; Ellison 2024; Singer et al. 2023; Natri et al. 2023), call for measurement not only of symptoms or cognitive ability but also of functional abilities and outcomes in autism, in both clinical practice and research (Tajik‐Parvinchi et al. 2023). These considerations and the proposed sub‐category of “profound autism” (Lord et al. 2022; Hughes et al. 2023) have brought to the forefront the need for additional methods to describe and monitor trends and functional impacts of autism that go beyond a single test score (e.g., IQ) or label (e.g., “high‐functioning”) (Furnier et al. 2024).

A promising approach to describing level of functioning is to use standard and well‐validated measures of adaptive functioning. Adaptive tests assess the age‐ and culturally‐appropriate skills used to independently navigate everyday life situations and social interactions and encompass a range of skill domains including social (e.g., interpersonal skills), communication (e.g., expressive and receptive language), daily living skills (e.g., safety, self‐care), and, for younger children, motor (e.g., fine and gross) (Tassé et al. 2012; Oakland and Daley 2013; American Association on Intellectual and Developmental Disabilities, n.d.). These measures offer additional insight into the types and levels of support an individual may require in order to achieve optimal outcomes in their environment and may help identify specific areas in which improved societal supports are needed. Limitations in adaptive functioning are an important component of intellectual disability (American Psychiatric Association 2013; American Association on Intellectual and Developmental Disabilities, n.d.). Adaptive challenges are also common in autistic children, ranging from mild to profound and requiring levels of support ranging from minimal to intensive; for example, a child with mild challenges may be able to learn and perform daily self‐care activities like dressing or feeding themselves with minimal support while an individual with profound challenges may need 24/7 care and assistance (American Psychiatric Association 2013; Tassé et al. 2012; Oakland and Daley 2013). Adaptive scores may provide more insight into practical, everyday support levels than IQ scores alone and into the rise in autism prevalence over past decades. Our goal was to use data available from 2000 to 2016 to describe trends over time in the prevalence of autism stratified by: (a) levels of adaptive functioning; and (b) the presence or absence of co‐occurring ID. We hypothesized, in line with previous research suggesting that much of the rise in autism prevalence has been among those without significant cognitive impairment (Autism and Developmental Disabilities Monitoring Network Surveillance Year 2000 Principal Investigators, Centers for Disease Control and Prevention 2007; Autism and Developmental Disabilities Monitoring Network Surveillance Year 2002 Principal Investigators, Centers for Disease Control and Prevention 2007; Autism and Developmental Disabilities Monitoring Network Surveillance Year 2004 Principal Investigators, Centers for Disease Control and Prevention 2009; Autism and Developmental Disabilities Monitoring Network Surveillance Year 2006 Principal Investigators, Centers for Disease Control and Prevention 2009; Autism and Developmental Disabilities Monitoring Network Surveillance Year 2008 Principal Investigators, Centers for Disease Control and Prevention 2012; Developmental Disabilities Monitoring Network Surveillance Year 2010 Principal Investigators, Centers for Disease Control and Prevention 2014; Christensen et al. 2018; Baio et al. 2018; Maenner et al. 2020; Croen et al. 2002; Van Naarden Braun et al. 2015; Thomas et al. 2017; Kogan et al. 2018), that we would find:

1. The prevalence of autism increased at each level of adaptive functioning, but the increase would be greater for autism with mild or no significant adaptive challenges than for autism with moderate to profound challenges.
2. The prevalence of autism with and without co‐occurring ID increased during the study period, but the increase would be greater for autism without co‐occurring ID.

## 2 Methods

### 2.1 Study Sample

ADDM is a CDC surveillance system that provides estimates of the prevalence of autism in 8‐year‐old children in the United States biennially. Between 2000 and 2016, sites throughout the US reviewed health, educational, and administrative records of children residing within the designated geographic study area. Records which noted an autism diagnosis or contained descriptions of behaviors potentially consistent with autism were reviewed by clinicians to determine whether the child met the surveillance case definition based on the DSM, Fourth Edition, Text Revision (DSM‐IV‐TR) (American Psychiatric Association 2000) and DSM‐5 (American Psychiatric Association 2013). Importantly, because of the systematic clinician review of diagnostic and behavioral descriptions in children's records, children with no previous diagnosis of autism but considered to meet DSM criteria for autism were included in the ADDM case counts, while other children with a previous diagnosis in their records were excluded if there was insufficient information in the records for the clinician reviewers to determine that DSM criteria for autism were met. Further details on the ADDM Network methodology for autism surveillance for the period 2000–2016, its validation and prevalence estimation approach can be found in previous publications (Autism and Developmental Disabilities Monitoring Network Surveillance Year 2000 Principal Investigators, Centers for Disease Control and Prevention 2007; Autism and Developmental Disabilities Monitoring Network Surveillance Year 2002 Principal Investigators, Centers for Disease Control and Prevention 2007; Autism and Developmental Disabilities Monitoring Network Surveillance Year 2004 Principal Investigators, Centers for Disease Control and Prevention 2009; Autism and Developmental Disabilities Monitoring Network Surveillance Year 2006 Principal Investigators, Centers for Disease Control and Prevention 2009; Autism and Developmental Disabilities Monitoring Network Surveillance Year 2008 Principal Investigators, Centers for Disease Control and Prevention 2012; Developmental Disabilities Monitoring Network Surveillance Year 2010 Principal Investigators, Centers for Disease Control and Prevention 2014; Christensen et al. 2018; Baio et al. 2018; Maenner et al. 2020; Van Naarden Braun et al. 2007). Additional information, including sociodemographic characteristics and results from IQ and adaptive tests, was also collected for each child. We limited our study sample to 8‐year‐old children from sites included in ADDM Morbidity and Mortality Weekly Report (MMWR) co‐occurring ID estimates, as determined by study year‐specific thresholds for the percentage of children with an IQ score recorded, resulting in a total sample size of 24,669 8‐year‐old children who met ADDM ASD criteria (Tables 1 and 2). Participating sites functioned as public health authorities under the Health Insurance Portability and Accountability Act of 1996 Privacy Rule in accordance with 45 CFR 46 and as authorized representatives of Individuals with Disabilities Education Act (IDEA) agencies to access education records under Family Education Rights and Privacy Act and IDEA consistent with 34 CFR Section 99.35. Sites met all applicable local institutional review board, privacy, and confidentiality requirements.

**TABLE 1:** Summary of cognitive ability estimates from the Autism and Developmental Disabilities Monitoring Network (ADDM), 2000–2016.

Study year	Threshold for site inclusion^a^	Study area included	Total cases^b^, ^c^	Total 8‐year‐olds^b^	% with IQ data^c^	% with IQ ≤ 70^c^	% with adaptive data^d^
2000 (Autism and Developmental Disabilities Monitoring Network Surveillance Year 2000 Principal Investigators, Centers for Disease Control and Prevention 2007)	> 85%	Arizona, Georgia, South Carolina	735	113,450	89%–94%^e^	40%–62%^e^	76.5%
2002 (Autism and Developmental Disabilities Monitoring Network Surveillance Year 2002 Principal Investigators, Centers for Disease Control and Prevention 2007)	≥ 80%	Arizona, Arkansas, Colorado, Georgia, North Carolina, South Carolina, Utah	1404	206,928	91.2%^d^	44.6%	74.0%
2004 (Autism and Developmental Disabilities Monitoring Network Surveillance Year 2004 Principal Investigators, Centers for Disease Control and Prevention 2009)	≥ 70%	Alabama, Arizona, Georgia, North Carolina, South Carolina^f^	882	113,072	92.6%^d^	43.8%	81.2%
2006 (Autism and Developmental Disabilities Monitoring Network Surveillance Year 2006 Principal Investigators, Centers for Disease Control and Prevention 2009)	≥ 70%	Alabama, Arizona, Colorado, Georgia, North Carolina, South Carolina	1670	175,457	89.2%^d^	41%	76.2%
2008 (Autism and Developmental Disabilities Monitoring Network Surveillance Year 2008 Principal Investigators, Centers for Disease Control and Prevention 2012)	≥ 70%	Arizona, Arkansas, Georgia, New Jersey, North Carolina, South Carolina, Utah	2139	157,855	92.6%	38%	77.7%
2010 (Developmental Disabilities Monitoring Network Surveillance Year 2010 Principal Investigators, Centers for Disease Control and Prevention 2014)	≥ 70%	Arkansas, Arizona, Georgia, Maryland, New Jersey, North Carolina, Utah	4140	242,120	87%	31%	64.7%
2012 (Christensen et al. 2018)	≥ 70%	Arizona, Arkansas, Colorado, Georgia, Maryland^g^, New Jersey, North Carolina, South Carolina, Utah	4234	267,398	80%	31.6%	59.7%
2014 (Baio et al. 2018)	≥ 70%	Arizona, Arkansas, Colorado, Georgia, Maryland, Minnesota, New Jersey, North Carolina, Tennessee	4623	265,113	80.3%	31%	58.7%
2016 (Maenner et al. 2020)	≥ 60%	Arizona, Arkansas, Colorado, Georgia, Maryland, Minnesota, New Jersey, North Carolina, Tennessee, Wisconsin	4895	259,784	79.6%	33.4%	61.8%

Abbreviations: ADDM, Autism and Developmental Disability Monitoring Network; IQ, intelligence quotient; MMWR, Morbidity and Mortality Weekly Report.

^a^ The percentage of children with a recorded intelligence test score required for site inclusion in ADDM MMWR estimate of co‐occurring intellectual disability for that study year.

^b^ Totals limited to sites included in MMWR estimates of cognitive ability; the total case numbers reported in this study may differ slightly from those reported in the corresponding year's MMWR due to disqualifications identified after MMWR publication.

^c^ Reported in MMWR.

^d^ Not reported in MMWR, calculated from the data.

^e^ Range among study sites are given for study year 2000 because no overall estimate was available; average percentage IQ data: 91%; average percentage with IQ ≤ 70: 49%.

^f^ Sites included in cognitive ability estimates were not specified in the MMWR; sites listed were identified from the data.

^g^ In 2012, a subset of 8‐year‐olds in Maryland had only health records for review and were not included in the cognitive ability estimates for that year.

**TABLE 2:** Child and sociodemographic characteristics of the study sample, by study year, Autism and Developmental Disabilities Monitoring Network, 2000–2016.

Child and sociodemographic characteristic	Study year								
	2000	2002	2004	2006	2008	2010	2012	2014	2016
	*n* (%)	*n* (%)	*n* (%)	*n* (%)	*n* (%)	*n* (%)	*n* (%)	*n* (%)	*n* (%)
Total	735	1402	882	1670	2139	4137	4189	4620	4895
Both IQ and adaptive score recorded	532 (72.4)	996 (71.0)	696 (78.9)	1225 (73.4)	1616 (75.5)	2562 (61.9)	2305 (55.0)	2524 (54.6)	2793 (57.1)
Child's sex									
Boys	581 (79.0)	1158 (82.6)	726 (82.3)	1369 (82.0)	1786 (83.5)	3412 (82.5)	3441 (82.1)	3744 (81.0)	4005 (81.8)
Girls	154 (21.0)	244 (17.4)	156 (17.7)	301 (18.0)	353 (16.5)	725 (17.5)	748 (17.9)	876 (19.0)	890 (18.2)
Child's race and ethnicity									
American Indian or Alaska Native, Non‐Hispanic (NH)	a	a	a	12 (0.7)	17 (0.8)	29 (0.7)	22 (0.5)	24 (0.5)	21 (0.4)
Asian or Pacific Islander, NH	16 (2.2)	20 (1.4)	28 (3.2)	49 (2.9)	81 (3.8)	161 (3.9)	177 (4.2)	199 (4.3)	268 (5.5)
Black, NH	184 (25.0)	304 (21.7)	272 (30.8)	402 (24.1)	567 (26.5)	789 (19.1)	855 (20.4)	1016 (22.0)	997 (20.4)
Hispanic	51 (6.9)	108 (7.7)	88 (10.0)	204 (12.2)	278 (13.0)	620 (15.0)	639 (15.3)	853 (18.5)	906 (18.5)
Other or Multiracial, NH	a	23 (1.6)	16 (1.8)	35 (2.1)	52 (2.4)	117 (2.8)	181 (4.3)	135 (2.9)	146 (3.0)
White, NH	430 (58.5)	914 (65.2)	452 (51.2)	913 (54.7)	1101 (51.5)	2381 (57.6)	2252 (53.8)	2287 (49.5)	2494 (50.9)
Missing	35 (4.8)	a	a	55 (3.3)	43 (2.0)	40 (1.0)	63 (1.5)	106 (2.3)	63 (1.3)
Median household income (census tract)									
Lowest tertile	120 (16.3)	280 (20.0)	206 (23.4)	438 (26.2)	541 (25.3)	1183 (28.6)	838 (20.0)	1276 (27.6)	1311 (26.8)
Middle	191 (26.0)	431 (30.7)	230 (26.1)	508 (30.4)	636 (29.7)	1458 (35.2)	1178 (28.1)	1540 (33.3)	1704 (34.8)
Highest tertile	269 (36.6)	551 (39.3)	328 (37.2)	523 (31.3)	698 (32.6)	1492 (36.1)	1412 (33.7)	1785 (38.6)	1876 (38.3)
Missing	155 (21.1)	140 (10.0)	118 (13.4)	201 (12.0)	264 (12.3)	4 (0.1)	761 (18.2)	19 (0.4)	4 (0.1)
Maternal education									
8th grade or less	19 (2.6)	23 (1.6)	19 (2.2)	30 (1.8)	43 (2.0)	104 (2.5)	100 (2.4)	116 (2.5)	418 (8.5)
High school	150 (20.4)	384 (27.4)	241 (27.3)	458 (27.4)	541 (25.3)	1185 (28.6)	1053 (25.1)	986 (21.3)	1235 (25.2)
College	153 (20.8)	381 (27.2)	262 (29.7)	533 (31.9)	668 (31.2)	1409 (34.1)	1288 (30.7)	998 (21.6)	1393 (28.5)
More than college	38 (5.2)	78 (5.6)	60 (6.8)	119 (7.1)	185 (8.6)	365 (8.8)	456 (10.9)	276 (6.0)	395 (8.1)
Missing	375 (51.0)	536 (38.2)	300 (34.0)	530 (31.7)	702 (32.8)	1074 (26.0)	1292 (30.8)	2244 (48.6)	1454 (29.7)
Age at earliest documented autism diagnosis									
0–2 years	66 (9.0)	145 (10.3)	97 (11.0)	216 (12.9)	346 (16.2)	705 (17.0)	814 (19.4)	813 (17.6)	903 (18.4)
Preschool (3–4 years)	131 (17.8)	325 (23.2)	183 (20.7)	353 (21.1)	433 (20.2)	1013 (24.5)	1063 (25.4)	1085 (23.5)	1263 (25.8)
Kindergarten (5 years)	41 (5.6)	140 (10.0)	92 (10.4)	164 (9.8)	206 (9.6)	461 (11.1)	423 (10.1)	473 (10.2)	511 (10.4)
1st or 2nd grade (6+ years)	91 (12.4)	254 (18.1)	151 (17.1)	263 (15.7)	371 (17.3)	722 (17.5)	711 (17.0)	760 (16.5)	893 (18.2)
No documented diagnosis	385 (52.4)	532 (37.9)	347 (39.3)	670 (40.1)	783 (36.6)	1236 (29.9)	1176 (28.1)	1487 (32.2)	1324 (27.0)
Missing	21 (2.9)	6 (0.4)	12 (1.4)	4 (0.2)	0 (0.0)	0 (0.0)	2 (0.0)	2 (0.0)	1 (0.0)
Source of abstracted records									
At least one school source	677 (92.1)	1212 (86.4)	729 (82.7)	1305 (78.1)	1857 (86.8)	3486 (84.3)	3039 (72.5)	3235 (70.0)	3273 (66.9)
Non‐school only	58 (7.9)	190 (13.6)	153 (17.3)	365 (21.9)	282 (13.2)	651 (15.7)	1150 (27.5)	1306 (28.3)	1587 (32.4)
Missing	0 (0.0)	0 (0.0)	0 (0.0)	0 (0.0)	0 (0.0)	0 (0.0)	0 (0.0)	79 (1.7)	35 (0.7)
Clinician reviewer's estimate of child's level of ASD‐associated impairment									
Low	173 (23.5)	248 (17.7)	251 (28.5)	701 (42.0)	777 (36.3)	1467 (35.5)	1334 (31.8)	1591 (34.4)	1863 (38.1)
High	547 (74.4)	528 (37.7)	311 (35.3)	969 (58.0)	1362 (63.7)	2670 (64.5)	2855 (68.2)	3027 (65.5)	3032 (61.9)
Missing	15 (2.0)	626 (44.7)	320 (36.3)	0 (0.0)	0 (0.0)	0 (0.0)	0 (0.0)	2 (0.0)	0 (0.0)
Number of autism discriminators (e.g., behaviors consistent with the core symptomatology of autism) described in child's records									
Lowest quartile	134 (18.2)	200 (14.3)	169 (19.2)	394 (23.6)	491 (23.0)	738 (17.8)	1133 (27.0)	1566 (33.9)	1017 (20.8)
2nd	224 (30.5)	304 (21.7)	216 (24.5)	577 (34.6)	661 (30.9)	1026 (24.8)	1078 (25.7)	1351 (29.2)	661 (13.5)
3rd	212 (28.8)	224 (16.0)	127 (14.4)	512 (30.7)	692 (32.4)	1365 (33.0)	1203 (28.7)	1218 (26.4)	531 (10.8)
Highest quartile	152 (20.7)	51 (3.6)	50 (5.7)	187 (11.2)	295 (13.8)	1008 (24.4)	775 (18.5)	483 (10.5)	242 (4.9)
Missing	13 (1.8)	326 (44.4)	320 (36.3)	0 (0.0)	0 (0.0)	0 (0.0)	0 (0.0)	2 (0.0)	2444 (49.9)
Number of associated features (e.g., emotional and behavioral features that often co‐occur with autism) described in child's records									
Lowest quartile	293 (39.9)	302 (21.5)	211 (23.9)	442 (26.5)	536 (25.1)	826 (20.0)	961 (22.9)	1246 (27.0)	1598 (32.6)
2nd	206 (28.0)	198 (14.1)	171 (19.4)	445 (26.6)	591 (27.6)	1052 (25.4)	1108 (26.5)	1205 (26.1)	1497 (30.6)
3rd	148 (20.1)	182 (13.0)	116 (13.2)	497 (29.8)	618 (28.9)	1245 (30.1)	1284 (30.7)	1327 (28.7)	1363 (27.8)
Highest quartile	75 (10.2)	97 (6.9)	64 (7.3)	286 (17.1)	394 (18.4)	1014 (24.5)	836 (20.0)	840 (18.2)	437 (8.9)
Missing	13 (1.8)	623 (44.4)	320 (36.3)	0 (0.0)	0 (0.0)	0 (0.0)	0 (0.0)	2 (0.0)	0 (0.0)

Abbreviations: ASD, autism spectrum disorder; DSM‐IV‐TR, Diagnostic and Statistical Manual of Mental Disorders, Fourth Edition, Text Revision; NH, non‐Hispanic.

^a^ Denotes that the cell value was suppressed to conceal frequencies less or equal to 10 to protect anonymity.

### 2.2 Measures of Intellectual Disability and Adaptive Level

Consistent with ADDM reporting practice, intellectual ability was assessed from a child's most recently administered IQ or other cognitive test. Adaptive functioning was measured using a Vineland Adaptive Behavior Scales (VABS); First (Sparrow et al. 1985) or Second (Sparrow et al. 2005) Edition or Social–Emotional Early Childhood Scales (Sparrow et al. 1998) score from a test administered between the ages of six to eight years (VABS 6–8 years).

We implemented multiple imputation (Woods et al. 2024) to impute missing adaptive and IQ scores using the *mice* (van Buuren and Groothuis‐Oudshoorn 2011) package in R version 4.3.2 (R Core Team, n.d.). Overall, adaptive and IQ scores were missing for 34.5% and 15.7% of autistic children, respectively. A detailed description of the imputation method is provided in the Supporting Information (Data S1: Supplementary Methods, Tables S1–S4, Figures S1–S4). We defined co‐occurring intellectual disability two ways: (1) IQ ≤ 70 (ADDM surveillance definition); and (2) IQ ≤ 70 and VABS 6–8 years score ≤ 70 (clinical definition). The latter is consistent with DSM and American Association on Intellectual and Developmental Disabilities definitions (American Psychiatric Association 2013; American Association on Intellectual and Developmental Disabilities, n.d.). Adaptive levels were determined based on VABS 6–8 years scores using the following cutoffs: moderate to profound adaptive challenges (< 50), mild (50–70), borderline (71–85), and none (> 85). The average age at most recent test administration for IQ was 71.8 months (standard deviation [SD]: 20.5) while that for any adaptive test was 67.3 (SD: 20.7). Among those with a VABS test between the ages of 6 to 8 years, the average age at testing was 86.1 (SD: 9.0). Among children who had both an IQ and any adaptive test recorded (*n* = 15,249), 74.7% had a gap of < 1 year between the two tests; for children with both an IQ and VABS 6–8 years test recorded (*n* = 3954), 86.3% had a gap of < 1 year between the two tests. The average gap between testing ages was 8.9 months (15.7) for any adaptive test and 4.8 months (11.6) for VABS 6–8 years test.

### 2.3 Data Analysis

We estimated the prevalence of autism (with 95% Wilson score confidence intervals [CI]) by adaptive level and co‐occurring ID status, based on both ADDM surveillance and clinical ID definitions, between 2000 and 2016, overall and stratified by child's sex using the formulas described in Lott and Reiter (2020). Denominators were obtained from census‐based population estimates of 8‐year‐old children residing in the geographic study area described in ADDM surveillance reports.

To explore the relationship between prevalence and child's sex, prevalence ratios (PRs) with 95% CIs were estimated using log‐binomial regression in the PROC GENMOD procedure in SAS Version 9.4 (Spiegelman and Hertzmark 2005; SAS Institute Inc 2013). The adaptive categories “borderline” and “none” were combined when computing PRs to avoid small cell sizes and improve statistical precision. PROC MIANALYZE was used to pool results from the imputed datasets and account for both within‐ and between‐imputation variance. We also conducted sensitivity analyses to assess the robustness of our results, including imputing any adaptive score, regardless of test or age, limiting analyses to sites that participated in at least eight of the nine study years (Arizona, Georgia, North Carolina), and among complete cases, limiting analyses to children with < 1 year between administration of their adaptive and IQ tests.

## 3 Results

### 3.1 Autism Prevalence by Adaptive Level, 2000–2016

**FIGURE 1:**
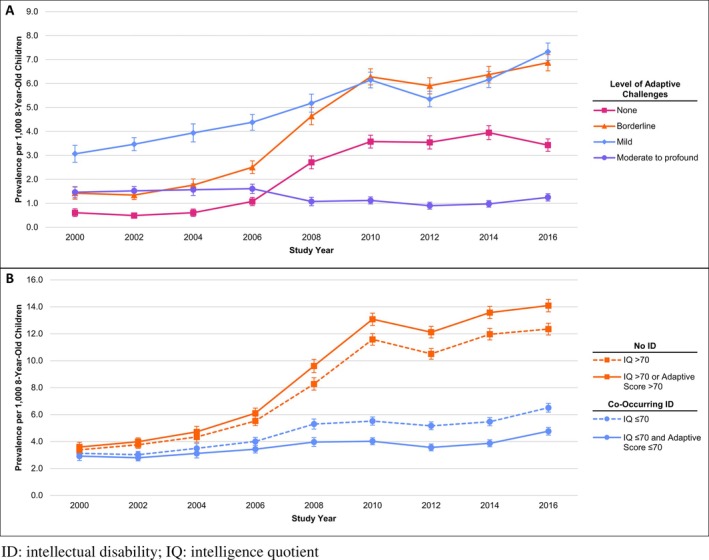
Prevalence of autism per 1000 8‐year‐old children (with 95% confidence intervals) by (A) level of adaptive challenges and (B) co‐occurring intellectual disability (ID) status, Autism and Developmental Disabilities Monitoring Network, 2000–2016. ID, intellectual disability; IQ, intelligence quotient.

In 2000, autism prevalence was highest in those with mild adaptive challenges (3.1 per 1000 8‐year‐old children [95% CI: 2.7–3.4]) and lowest for those with no significant adaptive challenges (0.6 [95% CI: 0.4–0.8]). By 2016, the prevalence of autism with no significant adaptive challenges had increased to 3.4 (95% CI: 3.2–3.7), a 464% increase compared to study year 2000, while the prevalence of autism with borderline adaptive challenges increased by 382% (from 1.4 to 6.9 per 1000) and the prevalence of autism with mild adaptive challenges increased by 139% (from 3.1 to 7.3 per 1000; Figure 1A). In contrast, the prevalence of autism with moderate to profound adaptive challenges was slightly lower in 2016 (1.2 [95% CI: 1.1–1.4]) than in 2000 (1.5 [95% CI: 1.2–1.7]).

**FIGURE 2:**
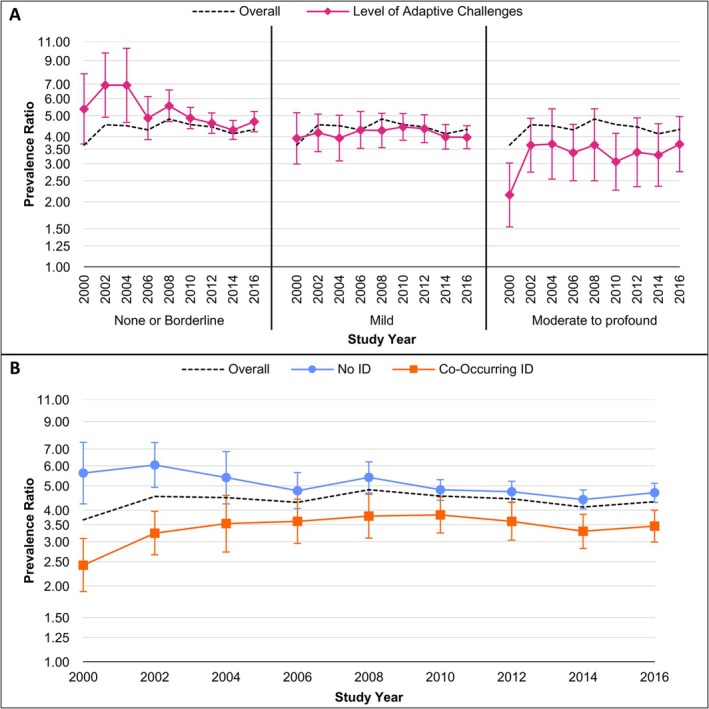
Male‐to‐female prevalence ratios comparing prevalence of autism by: (A) three levels of adaptive challenges; and (B) co‐occurring intellectual disability (ID) status based on intelligence quotient and adaptive score. All prevalence ratios are significantly greater than one (*p* < 0.05).

This general pattern was the same in boys and girls with the largest relative increases in autism with no significant adaptive challenges, followed by borderline and mild challenges, and little change or relative decreases in the prevalence of autism with moderate to profound adaptive challenges (Table 3). Male‐to‐female PRs were significantly greater than one at every adaptive level but tended to decrease with increasing levels of adaptive challenges (Figure 2A).

**TABLE 3:** Prevalence of autism per 1000 8‐year‐old children (with 95% confidence intervals) by adaptive level, Autism and Developmental Disabilities Monitoring Network, 2000–2016.

Level of adaptive challenges	Study year								
	2000	2002	2004	2006	2008	2010	2012	2014	2016
Boys									
None	1.1 (0.8–1.4)	0.9 (0.7–1.1)	1.0 (0.7–1.3)	1.8 (1.5–2.1)	4.5 (4.0–5.0)	5.9 (5.5–6.4)	5.7 (5.3–6.2)	6.3 (5.8–6.7)	5.7 (5.2–6.1)
Borderline	2.3 (1.9–2.8)	2.3 (2.0–2.6)	3.1 (2.6–3.5)	4.1 (3.6–4.6)	7.7 (7.1–8.4)	10.2 (9.6–10.8)	9.6 (9.0–10.2)	10.3 (9.7–10.9)	11.1 (10.5–11.7)
Mild	4.9 (4.2–5.5)	5.5 (5.0–6.0)	6.2 (5.6–6.9)	7.0 (6.4–7.6)	8.3 (7.6–8.9)	9.9 (9.4–10.5)	8.6 (8.0–9.2)	9.8 (9.2–10.3)	11.6 (10.9–12.2)
Moderate to profound	2.0 (1.6–2.4)	2.4 (2.1–2.7)	2.5 (2.0–2.9)	2.4 (2.1–2.8)	1.7 (1.4–2.0)	1.7 (1.4–1.9)	1.4 (1.1–1.6)	1.5 (1.3–1.7)	1.9 (1.7–2.2)
Girls									
None	0.2 (0.1–0.3)	0.1 (0.0–0.2)	0.2 (0.1–0.3)	0.4 (0.2–0.5)	0.8 (0.6–1.0)	1.1 (0.9–1.4)	1.3 (1.1–1.5)	1.6 (1.3–1.8)	1.1 (0.9–1.3)
Borderline	0.5 (0.3–0.7)	0.4 (0.2–0.5)	0.5 (0.3–0.6)	0.9 (0.7–1.1)	1.4 (1.1–1.7)	2.2 (1.9–2.5)	2.1 (1.8–2.4)	2.3 (2.0–2.6)	2.5 (2.2–2.8)
Mild	1.3 (1.0–1.6)	1.3 (1.1–1.6)	1.6 (1.3–2.0)	1.6 (1.4–1.9)	2.0 (1.6–2.3)	2.3 (2.0–2.5)	2.0 (1.7–2.3)	2.5 (2.2–2.7)	2.9 (2.6–3.2)
Moderate to profound	0.9 (0.7–1.2)	0.7 (0.5–0.8)	0.7 (0.5–0.9)	0.7 (0.6–0.9)	0.5 (0.3–0.6)	0.6 (0.4–0.7)	0.4 (0.3–0.6)	0.5 (0.3–0.6)	0.5 (0.4–0.7)

The patterns seen in analyses based on multiply imputed data were similar to those in complete case analyses (Tables S5 and S6), when any adaptive score was imputed, regardless of test or age (Tables S7 and S8), and when analyses were limited to sites that participated in at least eight of the nine study years (Tables S9 and S10).

### 3.2 Autism Prevalence by Co‐Occurring ID Status, 2000–2016

The prevalence of autism with and without co‐occurring ID increased between 2000 and 2016, with some variation year to year, regardless of ID definition used (Figure 1B). The relative increase in prevalence over the study period was larger in those without co‐occurring ID than in those with co‐occurring ID, regardless of which definition of ID was used (Figure 1B). This pattern was generally similar in boys and girls (Table 4). Boys had higher prevalence, both with and without co‐occurring ID, than girls for every study year, but the male‐to‐female PR was higher in those without co‐occurring ID (Figure 2B). These patterns were similar in complete case analyses (Tables S5 and S6), when we imputed any adaptive score, regardless of test or age (Tables S7 and S8), and when analyses were limited to sites that participated in at least eight of the nine study years (Tables S9 and S10).

**TABLE 4:** Prevalence of autism per 1000 8‐year‐old children (with 95% confidence intervals) with and without co‐occurring intellectual disability (ID), Autism and Developmental Disabilities Monitoring Network, 2000–2016.

Co‐occurring ID status	Study year								
	2000	2002	2004	2006	2008	2010	2012	2014	2016
ADDM surveillance definition of ID (IQ ≤ 70)									
Boys									
ID	4.4 (3.8–4.9)	4.6 (4.2–5.0)	5.4 (4.8–6.0)	6.2 (5.7–6.7)	8.3 (7.7–9.0)	8.7 (8.2–9.2)	8.0 (7.5–8.5)	8.4 (7.8–8.9)	10.1 (9.5–10.6)
No ID	5.8 (5.1–6.4)	6.4 (5.9–6.9)	7.3 (6.6–8.0)	9.0 (8.4–9.7)	13.8 (13.0–14.7)	19.1 (18.3–19.8)	17.3 (16.6–18.0)	19.4 (18.6–20.1)	20.2 (19.4–21.0)
Girls									
ID	1.9 (1.5–2.2)	1.4 (1.2–1.7)	1.5 (1.2–1.9)	1.7 (1.5–2)	2.2 (1.8–2.5)	2.3 (2.0–2.5)	2.2 (2.0–2.5)	2.5 (2.2–2.8)	2.8 (2.5–3.1)
No ID	1.0 (0.7–1.2)	1.0 (0.8–1.2)	1.3 (1–1.6)	1.8 (1.5–2.1)	2.5 (2.1–2.8)	3.9 (3.5–4.2)	3.5 (3.2–3.8)	4.3 (3.9–4.6)	4.2 (3.8–4.6)
Clinical definition of ID (both IQ and VABS composite scores ≤ 70)									
Boys									
ID	4.1 (3.6–4.6)	4.2 (3.8–4.6)	4.8 (4.2–5.4)	5.3 (4.8–5.8)	6.2 (5.6–6.8)	6.3 (5.9–6.8)	5.5 (5.1–6.0)	5.9 (5.5–6.3)	7.3 (6.8–7.8)
No ID	6.0 (5.4–6.7)	6.8 (6.3–7.2)	7.9 (7.1–8.6)	9.9 (9.3–10.6)	16.0 (15.1–16.8)	21.4 (20.6–22.2)	19.8 (19.0–20.5)	21.9 (21.1–22.7)	22.9 (22.1–23.8)
Girls									
ID	1.7 (1.4–2.1)	1.3 (1.1–1.5)	1.4 (1.1–1.7)	1.5 (1.2–1.8)	1.7 (1.4–1.9)	1.7 (1.4–1.9)	1.5 (1.3–1.8)	1.8 (1.5–2.0)	2.1 (1.9–2.4)
No ID	1.1 (0.8–1.4)	1.1 (0.9–1.3)	1.5 (1.2–1.8)	2.1 (1.8–2.4)	3.0 (2.6–3.4)	4.5 (4.1–4.8)	4.2 (3.8–4.6)	5.0 (4.6–5.4)	4.9 (4.5–5.3)

Abbreviations: ID, intellectual disability; IQ, intelligence quotient.

## 4 Discussion

This is the first study to describe trends over time in population‐level prevalence of autism by adaptive level and by co‐occurrence of ID with incorporation of an adaptive criterion. Our finding that the rise in autism prevalence between 2000 and 2016 was disproportionately in those with mild or no significant adaptive or intellectual limitations is consistent with the possibility that the overall rise in prevalence during the study period was due to improved detection of autism with milder phenotypes. It is also consistent with previous research suggesting that, as ASD prevalence has risen, the proportion with co‐occurring ID has decreased (Autism and Developmental Disabilities Monitoring Network Surveillance Year 2000 Principal Investigators, Centers for Disease Control and Prevention 2007; Autism and Developmental Disabilities Monitoring Network Surveillance Year 2002 Principal Investigators, Centers for Disease Control and Prevention 2007; Autism and Developmental Disabilities Monitoring Network Surveillance Year 2004 Principal Investigators, Centers for Disease Control and Prevention 2009; Autism and Developmental Disabilities Monitoring Network Surveillance Year 2006 Principal Investigators, Centers for Disease Control and Prevention 2009; Autism and Developmental Disabilities Monitoring Network Surveillance Year 2008 Principal Investigators, Centers for Disease Control and Prevention 2012; Developmental Disabilities Monitoring Network Surveillance Year 2010 Principal Investigators, Centers for Disease Control and Prevention 2014; Christensen et al. 2018; Baio et al. 2018; Maenner et al. 2020; Croen et al. 2002; Van Naarden Braun et al. 2015; Thomas et al. 2017; Kogan et al. 2018).

It is possible that efforts to boost identification of autism in recent decades have resulted in improved detection of autism with less severe phenotypes. Programs like the CDC's Learn the Signs. Act Early., first introduced in 2004, seek to better prepare parents to monitor their child's development to identify potential delays or missed milestones and equip them to bring concerns to their pediatrician's attention (Abercrombie et al. 2022). Since 2007, the American Academy of Pediatrics has recommended universal screening for ASD at 18 and 24 months (American Academy of Pediatrics, American Academy of Pediatrics Committee on Children With Disabilities 2007). Similarly, programs like Healthy People 2020 and the Maternal and Child Health Block Grant Program have emphasized the importance of developmental screenings (Centers for Disease Control and Prevention 2020; Health Resources and Services Administration Maternal and Child Health Bureau, n.d.).

The decline in the proportion of autistic children with significant adaptive limitations or co‐occurring ID over time could also be a result of improved access to early intervention services. The Individuals with Disabilities Education Act was amended in 1986, with further improvements introduced in 2004 and 2011, to enhance provision of early intervention services to children with disabilities under the age of three and identification of eligible children in order to support child development (Dragoo 2024; US Department of Education 2011). Healthy People 2020 (Centers for Disease Control and Prevention 2020) included the proportion of autistic children receiving services before age four as an objective. By age eight, children's test scores may reflect the effects of these early intervention programs, which have been shown to improve both cognitive and adaptive skill development (Dawson et al. 2010; Eckes et al. 2023; Franz et al. 2022). Improved access may also help explain why we saw decreases in the prevalence of autism with moderate to profound adaptive challenges over the study period. We found both the percentage of children with a documented diagnosis in their records and the percentage of children diagnosed in preschool or before increased over the study period. It is possible that due to improved early detection, more children with significant adaptive support needs may have benefited from intervention‐related boosts in adaptive skill development.

While in the earlier years, the prevalence of autism with co‐occurring ID based on the surveillance definition of ID (i.e., IQ ≤ 70) was similar to that based on the clinical definition of ID (i.e., IQ ≤ 70 and significant limitations in adaptive functioning), the difference between the two estimates grew larger over the study period. With increasing identification of autism with milder presentations, it will be important to incorporate adaptive scores to correctly characterize the prevalence of autism with co‐occurring ID. Additionally, adaptive scores allow estimation of ID severity or level of support needed that is consistent with the DSM‐5 criteria for ID severity levels (American Psychiatric Association 2013), and provide more granular descriptions of functioning and support needs than the use of IQ scores alone.

A large male‐to‐female ratio is seen consistently in epidemiologic studies of autism (Autism and Developmental Disabilities Monitoring Network Surveillance Year 2000 Principal Investigators, Centers for Disease Control and Prevention 2007; Autism and Developmental Disabilities Monitoring Network Surveillance Year 2002 Principal Investigators, Centers for Disease Control and Prevention 2007; Autism and Developmental Disabilities Monitoring Network Surveillance Year 2004 Principal Investigators, Centers for Disease Control and Prevention 2009; Autism and Developmental Disabilities Monitoring Network Surveillance Year 2006 Principal Investigators, Centers for Disease Control and Prevention 2009; Autism and Developmental Disabilities Monitoring Network Surveillance Year 2008 Principal Investigators, Centers for Disease Control and Prevention 2012; Developmental Disabilities Monitoring Network Surveillance Year 2010 Principal Investigators, Centers for Disease Control and Prevention 2014; Christensen et al. 2018; Baio et al. 2018; Maenner et al. 2020; Zeidan et al. 2022; Loomes et al. 2017; Nicholas et al. 2008; Posserud et al. 2021; Fombonne 2005), with a ratio of approximately 4:1 commonly cited (American Psychiatric Association 2013) but with notable variability across studies (Zeidan et al. 2022; Loomes et al. 2017; Fombonne 2005). However, past research suggests that this ratio may differ depending on cognitive level, with a more pronounced male‐to‐female ratio among autistic individuals with IQs > 70 (Loomes et al. 2017; Nicholas et al. 2008; Posserud et al. 2021; Fombonne 2005; Cruz et al. 2025). Researchers have theorized that this effect may be indicative of biases in the autism diagnostic process, including potential biases in standard autism diagnostic instruments like the ADOS (Cruz et al. 2025), leading to late diagnosis and under‐identification of autistic females who do not exhibit significant difficulties in multiple areas of functioning (Loomes et al. 2017; Posserud et al. 2021; Cruz et al. 2025). Consistent with past research, we found the male‐to‐female PR to be most pronounced in those without co‐occurring ID or significant adaptive challenges (Loomes et al. 2017; Nicholas et al. 2008; Posserud et al. 2021). Of note, while the overall male‐to‐female PR was relatively stable over the study period, we saw a downward trend over time in this ratio in those without co‐occurring ID or significant adaptive challenges. However, the male‐to‐female PR in those with ID and moderate to profound adaptive challenges trended upwards over the same time period, suggesting that the well‐documented male‐to‐female ratio may not be entirely explained by under‐identification of females with milder presentations.

### 4.1 Limitations

While there was adaptive test information available for nearly two‐thirds of our study sample, there remained a large amount of missing adaptive information, and 16% of children were also missing IQ scores. To mitigate potential ascertainment bias, we implemented multiple imputation, for which a key assumption is that the data are missing at random. While we cannot formally test this assumption, we observed similar trends and patterns in complete case and supplementary analyses (see Tables S5–S10).

Additionally, in the absence of domain‐level adaptive scores, we were unable to examine an ID definition requiring limitations in only one adaptive domain (American Psychiatric Association 2013; Perry et al. 2009). Our reliance on composite adaptive scores may have led to underestimation of the proportion of autistic children who met ID criteria. Because the core symptoms of autism relate to social communication, autistic children may have relative strengths in conceptual and practical adaptive domains accompanied by significant support needs in socialization (Perry et al. 2009; Yang et al. 2016).

Some variation across the study period may be due to surveillance protocol changes. For three of the surveillance years (2002, 2004, and 2016), a child could meet ASD criteria if they had a documented diagnosis, regardless of whether clinicians determined that there were sufficient descriptions of ASD‐related behaviors in the child's records, and the 2016 case definition was based on DSM‐5 whereas in earlier years it was based on DSM‐IV criteria (Autism and Developmental Disabilities Monitoring Network Surveillance Year 2000 Principal Investigators, Centers for Disease Control and Prevention 2007; Autism and Developmental Disabilities Monitoring Network Surveillance Year 2002 Principal Investigators, Centers for Disease Control and Prevention 2007; Autism and Developmental Disabilities Monitoring Network Surveillance Year 2004 Principal Investigators, Centers for Disease Control and Prevention 2009; Autism and Developmental Disabilities Monitoring Network Surveillance Year 2006 Principal Investigators, Centers for Disease Control and Prevention 2009; Autism and Developmental Disabilities Monitoring Network Surveillance Year 2008 Principal Investigators, Centers for Disease Control and Prevention 2012; Developmental Disabilities Monitoring Network Surveillance Year 2010 Principal Investigators, Centers for Disease Control and Prevention 2014; Christensen et al. 2018; Baio et al. 2018; Maenner et al. 2020).

Other limitations stem from the study's reliance on real‐world public health surveillance data. Year‐to‐year variations in study site composition could have contributed to the prevalence trends observed, as there were substantial differences between sites in not only overall prevalence but also in case characteristics, such as ASD subtype (Autism and Developmental Disabilities Monitoring Network Surveillance Year 2000 Principal Investigators, Centers for Disease Control and Prevention 2007; Autism and Developmental Disabilities Monitoring Network Surveillance Year 2002 Principal Investigators, Centers for Disease Control and Prevention 2007; Autism and Developmental Disabilities Monitoring Network Surveillance Year 2004 Principal Investigators, Centers for Disease Control and Prevention 2009; Autism and Developmental Disabilities Monitoring Network Surveillance Year 2006 Principal Investigators, Centers for Disease Control and Prevention 2009; Autism and Developmental Disabilities Monitoring Network Surveillance Year 2008 Principal Investigators, Centers for Disease Control and Prevention 2012; Developmental Disabilities Monitoring Network Surveillance Year 2010 Principal Investigators, Centers for Disease Control and Prevention 2014; Christensen et al. 2018; Baio et al. 2018; Maenner et al. 2020). Because the makeup of ADDM varied somewhat across years, differential site participation could have influenced the results. However, we found a similar pattern when we limited our analyses to the three sites that participated in at least eight of the nine study periods (Tables S9 and S10).

Finally, while ADDM is population‐based, sites were selected through a competitive process based on their ability to conduct surveillance, and our sample was restricted to ADDM sites with IQ data recorded for a certain percentage of autistic children. Therefore, the large and diverse population‐based sample included in this study is not necessarily representative of the general US population of 8‐year‐old children.

## 5 Conclusions

Using adaptive scores available in health and education records, we estimated the population‐based prevalence of autism by adaptive level and co‐occurring ID status. The increase in autism prevalence between 2000 and 2016 was disproportionately among those with mild or no significant adaptive limitations and without co‐occurring ID. These trends could indicate improved identification of autistic children with milder phenotypes over time as well as improvements in functioning due to increased access to effective therapies. This study demonstrates the importance of incorporating adaptive behavior scores in epidemiologic studies of autism to better identify the strengths and support needs of this population. Future research could examine ongoing ADDM surveillance data to assess whether the trends we observed have continued.

## Funding

This study was supported by the Centers for Disease Control and Prevention grant 1NUR3DD000106, the Waisman Center (Eunice Kennedy Shriver National Institute of Child Health and Human Development grant number U54 HD090256), and the University of Wisconsin‐Madison Wisconsin Distinguished Graduate Fellowship. The findings and conclusions are solely the responsibility of the authors and do not necessarily represent the official views of the Centers for Disease Control and Prevention.

## Ethics Statement

Participating study sites functioned as public health authorities under the Health Insurance Portability and Accountability Act of 1996 Privacy Rule in accordance with 45 CFR 46 and as authorized representatives of Individuals with Disabilities Education Act (IDEA) agencies to access education records under Family Education Rights and Privacy Act and IDEA consistent with 34 CFR Section 99.35. Sites met all applicable local institutional review board, privacy, and confidentiality requirements.

## Conflicts of Interest

The authors declare no conflicts of interest.

## Supporting information


**Data S1:** aur70167‐sup‐0001‐SupplementaryMaterials1.docx.

**Data S2:** aur70167‐sup‐0002‐SupplementaryMaterials2.docx.

## Data Availability

Research data are not shared.
