# Supplementary material for: Trends Over Time in the Prevalence of Autism by Adaptive and Intellectual Functioning Levels
Source: Autism Res. 2025 Dec 28;19(1):e70167. doi: 10.1002/aur.70167 (PMC12853238; doi:10.1002/aur.70167)
Supplement: Supplementary file 1 — Data S1: aur70167‐sup‐0001‐SupplementaryMaterials1.docx. [file AUR-19-0-s002.docx]

**Supplementary Methods: Further Details on the Multiple Imputation Procedure**

**Contents**

[Supplementary Methods. Rationale and Detailed Description of the Imputation Procedure 2](#_Toc205388670)

[Supplementary Table 1. Child and sociodemographic characteristics of children with autism in the Autism and Developmental Disabilities Monitoring Network, 2000-2016, overall and by availability of adaptive and intelligence quotient test information 6](#_Toc205388671)

[Supplementary Table 2. Variables included in the multiple imputation model 10](#_Toc205388672)

[Supplementary Table 3. Conversion of categorical intellectual and adaptive classifications to standard scores prior to imputation 13](#_Toc205388673)

[Supplementary Table 4. Additional details about the multiple imputation procedure 13](#_Toc205388674)

[Supplementary Figure 1. Trace plots from the first step of the multiple imputation procedure during which adaptive test scores from non-Vineland Adaptive Behavior Scales (VABS) tests or obtained before age six were converted to VABS 6 to 8 years scores 14](#_Toc205388675)

[Supplementary Figure 2. Trace plots from the second step of the multiple imputation procedure during which VABS 6 to 8 years scores and any other missing variables were imputed 16](#_Toc205388676)

[Supplementary Figure 3. Comparison of observed and imputed values for numeric variables 19](#_Toc205388677)

[Supplementary Figure 4. Comparison of observed and imputed values for categorical variables 20](#_Toc205388678)

# Supplementary Methods. Multiple Imputation Rationale and Detailed Description of the Imputation Procedure

Missing data is a source of potential bias in many epidemiological studies. In the Autism and Developmental Disabilities Monitoring Network (ADDM), global adaptive and intelligence test standard scores are abstracted from children’s evaluations when available. If unavailable, other types of scores (e.g., age equivalent, ratio intelligence quotient [IQ]), classifications (e.g., clinician descriptions of functioning), or domain scores (e.g., nonverbal or performance IQ, verbal IQ) may be abstracted. However, among the 32,808 autism cases identified by ADDM between 2000 and 2016, 24.1% and 42.7% of children had no IQ or adaptive test result available, respectively. While historically, complete case analysis, or listwise deletion, was used to address missing data, this method decreases power and may introduce bias if there are systematic differences between participants for whom data is available and those whose data is incomplete or entirely missing. Additionally, because individuals from underrepresented racial, ethnic, and socioeconomic groups may have lower quality or fewer evaluations for abstraction, relying on listwise deletion may exclude these individuals from our analysis. In light of these limitations, we theorized that the use of multiple imputation for analysis of ADDM data would not only mitigate bias resulting from missing data but also potentially facilitate better representation in our study sample and improve the generalizability of our findings.^1-3^ Therefore, we chose to utilize multiple imputation through chained equations (mice) to account for missing test scores as well as demographic characteristics in our data.

A key assumption of multiple imputation methods is that the missing datapoints are ‘missing at random’ (MAR), meaning that after controlling for the observed data, the probability that a datapoint is missing is not dependent on the unobserved data.^1-2^ The more stringent assumption that the data is missing completely at random (MCAR) highly unlikely to be true in our data (Little’s MCAR test p<0.05^4^) but not required in multiple imputation. There were substantial study site differences in the percentage of children with recorded IQ (percent missing range: 64.6%% in Missouri to 6.7%% in North Carolina, average: 27.9%) and adaptive (percent missing range: 76.0% in Missouri to 13.0% in North Carolina, average 45.2%) scores, which may reflect different testing practices and state-specific assessment requirements for special education and government programs. Because site variability in IQ availability, ADDM Morbidity and Mortality Weekly Report (MMWR) surveillance reports for study years 2000 through 2016 limited cognitive ability estimates to sites that met a minimum threshold for the percentage of autistic children with IQ scores recorded (see Table 1 in the main text). To further increase the likelihood that the MAR assumption was met – and to facilitate comparisons to previously reported MMWR estimates – we also limited our analysis to the study sites included in ADDM MMWR surveillance report cognitive ability estimates for each study year (n=24,669; see Table 1 in the main text). Amongst this subset, 84.3% and 65.5% had an IQ or adaptive score recorded, respectively; 61.8% had both scores recorded (range: 78.9% in 2004 to 54.6% in 2014; average: 66.7%).

Both adaptive and IQ scores were less likely to be missing for children with records from school sources compared to those with only non-school sources, likely due to federal requirements regarding comprehensive evaluation – which may include both cognitive and adaptive testing – of children for determination of special education eligibility. See Supplementary Table 1 for additional information on factors related to adaptive and IQ score availability in ADDM. Other factors which may contribute to a child having test scores in their records include: undergoing developmental evaluations for suspected intellectual disability, autism, or other co-occurring conditions like anxiety, depression, or attention-deficit/hyperactivity disorder; the presence of other developmental concerns, such as language delays or a developmental plateau or regression; or testing to qualify for government assistance or other services. It may be expected that children who were not evaluated for one of the aforementioned reasons may have milder phenotypes; due to the nature of the relationships between adaptive functioning, cognitive functioning, autism severity, and co-occurring emotional and behavioral problems^5-7^, we might also expect that these children had better adaptive and intellectual functioning. Therefore, there may be differences between children with and without adaptive and IQ test scores recorded in their records. However, because ADDM collects a wealth of information, we were able to control for these potential missing mechanisms by including variables related to factors that may be associated with missing test scores in our imputation model (see Supplementary Table 2). Further, IQ scores and other important phenotypic measures (e.g., number of DSM-IV-TR criteria met, the number of autism-specific behaviors indicated in children’s records) were also available for most of the children in our sample and could be included to improve prediction of missing adaptive scores. Thus, we believe the MAR assumption to be reasonable for the missing adaptive and IQ scores in our ADDM data.

Unlike studies performed in a more controlled clinical or research setting, test scores in ADDM data come from a multitude of tests (including different test editions and forms, such as caregiver or teacher report) administered at different ages. To maximize comparability of adaptive scores, we selected scores from the most commonly administered test, the Vineland Adaptive Behavior Scales (VABS; including the first and second editions as well as the Vineland Social-Emotional Early Childhood Scales) from tests administered between the ages of six to eight years (VABS 6-8yrs) as our primary adaptive measure, and, prior to multiple imputation in the full dataset, we converted scores from other adaptive tests and ages to a VABS 6-8yrs score. Although IQ test results came from similarly varied tests and ages, ADDM surveillance reports estimate a child’s cognitive ability based on their most recent IQ score, regardless of test or age; thus, we followed this same strategy to measure IQ in our analyses.

For individuals with a non-standard score or clinician estimate of functional level recorded in place of a score, their non-standard scores or clinician estimate was converted to a score prior to selection of auxiliary variables for the imputation model and the imputation itself. See Supplementary Table 3 for additional details on this process. For children with multiple tests administered at the same age, we implemented a ‘benefit of the doubt’ approach and selected their highest score from that age, as is consistent with ADDM surveillance report methodology.

From a wider set of variables selected based on prior literature or hypothesized associations, we selected a subset of variables based on relevance to our analyses, theorized associations with missing variables based on substantive knowledge, or observed associations with either the missing status of adaptive or IQ scores or recorded adaptive and IQ scores. For the latter, variables were selected if they had (a) at least a 0.15 Spearman correlation with adaptive or IQ scores or their missing status and (b) an outflux value of at least 0.5. The final imputation model consisted of the primary outcomes of interest (i.e., IQ and VABS 6-8yrs score) and an additional 31 analytically relevant (i.e., child’s sex and race and ethnicity) and auxiliary variables.^8-10^ See Supplementary Table 2 for a full list of these variables.

We performed multiple imputation using the *mice* program^11^, version 3.16.0, in R version 4.3.2.^12^ The imputation procedure was performed in two steps. First, a single imputation was performed in the subset of children who had any adaptive score recorded (n=16,164) in order to convert other adaptive scores to a VABS 6-8yr scores. Following this, these imputed VABS 6-8yrs scores were incorporated into the full dataset (n=24,669), and relevant demographic characteristics, auxiliary variables, IQ, and, for children who had no adaptive test information recorded (n=8,505), VABS 6-8yrs scores were imputed. Thirty-five imputed datasets were generated; both VABS 6-8yrs and IQ scores were imputed using the Bayesian linear regression method. See Supplementary Table 4 for further details on the imputation model. Imputation checks were performed through examination of trace plots to assess algorithm convergence (Supplementary Figures 1-2) and comparisons of imputed values to observed values to determine whether the imputed values were plausible (Supplementary Figures 3-4).^13^ In the multiply imputed data, results from each imputed dataset were pooled using PROC MIANALYZE in SAS Version 9.4^14^ or using formulas for calculation of Wilson confidence intervals in multiply imputed data presented in Lott and Reiter.^15^

**Supplementary References**

1. Woods AD, Gerasimova D, Van Dusen B, et al. Best practices for addressing missing data through multiple imputation. *Infant Child Dev.* 2024;33(1):e2407. doi: 10.1002/icd.2407
2. Rubin DB. Inference and missing data. *Biometrika.* 1976;63(3):581-592. doi: 10.1093/biomet/63.3.581
3. Schafer JL. Multiple imputation: A primer. *Stat. Methods Med. Res.* 1999;8(1):3-15. doi: 10.1177/096228029900800102
4. Little RJ. A test of missing completely at random for multivariate data with missing values. *J Am Stat Assoc*. 1988;83(404):1198-1202.
5. Perry A, Flanagan HE, Geier JD, Freeman NL. Brief report: The Vineland Adaptive Behavior Scales in young children with autism spectrum disorders at different cognitive levels. *J Autism Dev Disord*. 2009;39(7):1066-78. doi: 10.1007/s10803-009-0704-9
6. Yang S, Paynter JM, Gilmore L. Vineland Adaptive Behavior Scales-II profile of young children with autism spectrum disorder. *J Autism Dev Disord*. 2016;46:64-73. doi: 10.1007/s10803-015-2543-1
7. Furnier SM, Ellis Weismer S, Rubenstein E, et al. Using adaptive behavior scores to convey level of functioning in children with autism spectrum disorder: Evidence from the Study to Explore Early Development. *Autism*. 2024;28(5):1135-1149. doi: 10.1177/13623613231193194
8. Sterne JA, White IR, Carlin JB, et al. Multiple imputation for missing data in epidemiological and clinical research: Potential and pitfalls. *BMJ*. 2009;338:b2393. doi: 10.1136/bmj.b2393
9. van Buuren S. 9.1 Too many columns. Flexible Imputation of Missing Data. Date unknown. Accessed October 31, 2024. https://stefvanbuuren.name/fimd/sec-toomany.html
10. Van Ginkel JR, Linting M, Rippe RC, Van Der Voort A. Rebutting existing misconceptions about multiple imputation as a method for handling missing data*. J. Pers. Assess.* 2020;102(3):297-3086. doi: 10.1080/00223891.2018.1530680
11. van Buuren S, Groothuis-Oudshoorn K. mice: multivariate imputation by chained equations in R. *J. Stat. Softw*. 2011;45(3):1-67. doi: 10.18637/jss.v045.i03
12. R: a language and environment for statistical computing (Version 4.3.2). Available at: https://www.R-project.org.
13. Nguyen CD, Carlin JB, Lee KJ. Model checking in multiple imputation: An overview and case study. *Emerg. Themes Epidemiol.* 2017;14:8. doi: 10.1186/s12982-017-0062-6
14. SAS Institute Inc. SAS (Version 9.4). SAS Institute; 2013
15. Lott A, Reiter JP. Wilson confidence intervals for binomial proportions with multiple imputation for missing data. *Am Stat.* 2020;74(2):109-115. doi: 10.1080/00031305.2018.1473796

Supplementary Table 1. Child and sociodemographic characteristics of children with autism in the Autism and Developmental Disabilities Monitoring Network, 2000-2016, overall and by availability of adaptive and intelligence quotient (IQ) test information

| **Characteristic** | **Overall** | **Adaptive Score Missing** | | | **IQ Score Missing** | | |
| --- | --- | --- | --- | --- | --- | --- | --- |
|  | **n**  **(Column %)** | **n**  **(Row %)** | **Prevalence Ratio**  **(95% Confidence Interval)^a^** | | **n (Row %)** | **Prevalence Ratio**  **(95% Confidence Interval)^a^** | |
|  |  |  | **Unadjusted** | **Adjusted^b^** |  | **Unadjusted** | **Adjusted^b^** |
| **Total** | 24669 | 8505 (34.5) | NA | NA | 3864 (15.7) | NA | NA |
| **Child’s sex** | | | | | | | |
| Boys | 20222 (82.0) | 7018 (34.7) | Reference | Reference | 3116 (15.4) | Reference | Reference |
| Girls | 4447 (18.0) | 1487 (33.4) | 0.96 (0.92, 1.01) | 0.96 (0.92, 1.00) | 748 (16.8) | **1.09 (1.01, 1.17)** | 1.07 (1.00, 1.15) |
| **Child’s race and ethnicity** | | | | | | | |
| American Indian or Alaska Native, Non-Hispanic (NH) | 143 (0.6) | 41 (28.7) | 0.79 (0.61, 1.02) | 0.88 (0.69, 1.13) | 16 (11.2) | 0.75 (0.47, 1.20) | 0.93 (0.59, 1.46) |
| Asian or Pacific Islander, NH | 999 (4.1) | 301 (30.1) | **0.83 (0.75, 0.91)** | **0.82 (0.75, 0.90)** | 171 (17.1) | 1.15 (1.00, 1.33) | 1.09 (0.95, 1.26) |
| Black, NH | 5386 (21.8) | 1454 (27.0) | **0.74 (0.71, 0.78)** | **0.82 (0.79, 0.86)** | 798 (14.8) | 1.00 (0.92, 1.08) | 1.06 (0.98, 1.14) |
| Hispanic | 3747 (15.2) | 1439 (38.4) | **1.06 (1.01, 1.11)** | **0.92 (0.88, 0.96)** | 652 (17.4) | **1.17 (1.08, 1.27)** | 1.00 (0.92, 1.08) |
| Other or Multiracial, NH | 714 (2.9) | 242 (33.9) | 0.93 (0.84, 1.04) | 0.91 (0.83, 1.00) | 124 (17.4) | 1.17 (0.99, 1.38) | 1.08 (0.92, 1.27) |
| White, NH | 13224 (53.6) | 4802 (36.3) | Reference | Reference | 1965 (14.9) | Reference | Reference |
| Missing | 456 (1.9) | 226 (49.6) | **1.36 (1.24, 1.50)** | **1.22 (1.14, 1.30)** | 138 (30.3) | **2.04 (1.76, 2.35)** | **1.77 (1.54, 2.03)** |
| **Median household income (census tract tertile)** | | | | | | | |
| Low | 6193 (25.1) | 1945 (31.4) | **0.86 (0.82, 0.90)** | **0.94 (0.90, 0.98)** | 969 (15.7) | 1.01 (0.94, 1.09) | **1.15 (1.07, 1.24)** |
| Middle | 7876 (31.9) | 2712 (34.4) | **0.94 (0.90, 0.98)** | 0.99 (0.95, 1.02) | 1201 (15.3) | 0.98 (0.92, 1.06) | 1.06 (0.99, 1.13) |
| High | 8934 (36.2) | 3273 (36.6) | Reference | Reference | 1386 (15.5) | Reference | Reference |
| Missing | 1666 (6.8) | 575 (34.5) | 0.94 (0.88, 1.01) | **1.23 (1.13, 1.34)** | 308 (18.5) | **1.19 (1.07, 1.33)** | **1.25 (1.04, 1.50)** |
| **Maternal education** | | | | | | | |
| 8^th^ grade or less | 872 (3.5) | 286 (32.8) | **0.87 (0.78, 0.97)** | 0.96 (0.86, 1.06) | 156 (17.9) | 1.12 (0.94, 1.33) | 0.93 (0.78, 1.12) |
| High school | 6233 (25.3) | 2201 (35.3) | 0.93 (0.87, 1.00) | 0.95 (0.90, 1.01) | 970 (15.6) | 0.97 (0.87, 1.09) | 1.01 (0.90, 1.13) |
| College | 7085 (28.7) | 2553 (35.3) | 0.95 (0.89, 1.01) | 0.99 (0.93, 1.04) | 1109 (15.7) | 0.98 (0.87, 1.10) | 1.01 (0.90, 1.12) |
| More than college | 1972 (8.0) | 747 (37.9) | Reference | Reference | 315 (16.0) | Reference | Reference |
| Missing | 8507 (34.5) | 2718 (32.0) | **0.84 (0.79, 0.90)** | 1.04 (0.98, 1.10) | 1314 (15.5) | 0.97 (0.86, 1.08) | 1.12 (1.00, 1.25) |
| **Age at earliest documented autism spectrum disorder (ASD) diagnosis** | | | | | | | |
| 0-2 yrs | 4105 (16.6) | 1219 (29.7) | Reference | Reference | 756 (18.4) | Reference | Reference |
| Preschool (3-4 yrs) | 5849 (23.7) | 1754 (30.0) | 1.01 (0.95, 1.07) | 1.00 (0.95, 1.06) | 1035 (17.7) | 0.96 (0.88, 1.05) | 0.95 (0.88, 1.03) |
| Kindergarten (5 yrs) | 2511 (10.2) | 816 (32.5) | **1.09 (1.02, 1.18)** | **1.11 (1.04, 1.18)** | 379 (15.1) | **0.82 (0.73, 0.92)** | **0.86 (0.77, 0.96)** |
| 1^st^/2^nd^ grade (6+ yrs) | 4216 (17.1) | 1714 (40.7) | **1.37 (1.29, 1.45)** | **1.39 (1.32, 1.46)** | 588 (14.0) | **0.76 (0.69, 0.84)** | **0.81 (0.74, 0.89)** |
| No documented diagnosis | 7940 (32.2) | 2992 (37.7) | **1.27 (1.20, 1.34)** | **1.39 (1.33, 1.46)** | 1098 (13.8) | **0.75 (0.69, 0.82)** | **0.89 (0.82, 0.97)** |
| Missing | 48 (0.2) | 10 (20.8) | 0.70 (0.40, 1.22) | 1.06 (0.61, 1.85) | 8 (16.7) | 0.91 (0.48, 1.71) | 1.39 (0.74, 2.62) |
| **Source of abstracted records** | | | | | | | |
| At least one school source | 18813 (76.3) | 5210 (27.7) | **0.49 (0.48, 0.51)** | **0.57 (0.55, 0.59)** | 1717 (9.1) | **0.25 (0.23, 0.26)** | **0.23 (0.22, 0.25)** |
| Non-school only | 5742 (23.3) | 3228 (56.2) | Reference | Reference | 2130 (37.1) | Reference | Reference |
| Missing | 114 (0.5) | 67 (58.8) | 1.05 (0.89, 1.22) | 0.91 (0.77, 1.07) | 17 (14.9) | **0.40 (0.26, 0.62)** | **0.36 (0.23, 0.56)** |
| **Clinician reviewer’s estimate of child’s level of ASD-associated impairment** | | | | | | | |
| Low | 8405 (34.1) | 3424 (40.7) | Reference | Reference | 1129 (13.4) | Reference | Reference |
| High | 15301 (62.0) | 4892 (32.0) | **0.78 (0.76, 0.81)** | **0.78 (0.76, 0.81)** | 2658 (17.4) | **1.29 (1.21, 1.38)** | **1.24 (1.16, 1.32)** |
| Missing | 963 (3.9) | 189 (19.6) | **0.48 (0.42, 0.55)** | **0.58 (0.49, 0.67)** | 77 (8.0) | **0.60 (0.48, 0.74)** | 1.11 (0.84, 1.48) |
| **Number of DSM-IV-TR criteria met** | | | | | | | |
| 1^st^ quartile | 6727 (27.3) | 3432 (51.0) | Reference | Reference | 1563 (23.2) | Reference | Reference |
| 2^nd^ | 6358 (25.8) | 2268 (35.7) | **0.70 (0.67, 0.73)** | **0.76 (0.73, 0.78)** | 991 (15.6) | **0.67 (0.62, 0.72)** | **0.69 (0.65, 0.74)** |
| 3^rd^ | 3983 (16.2) | 1015 (25.5) | **0.50 (0.47, 0.53)** | **0.57 (0.54, 0.60)** | 443 (11.1) | **0.48 (0.43, 0.53)** | **0.52 (0.47, 0.57)** |
| 4^th^ | 4197 (17.0) | 793 (18.9) | **0.37 (0.35, 0.40)** | **0.42 (0.40, 0.45)** | 389 (9.3) | **0.40 (0.36, 0.44)** | **0.43 (0.38, 0.47)** |
| Missing | 3404 (13.8) | 997 (29.3) | **0.57 (0.54, 0.61)** | **0.68 (0.64, 0.73)** | 478 (14.0) | **0.60 (0.55, 0.66)** | **0.61 (0.54, 0.68)** |
| **Number of autism discriminators indicated in records** | | | | | | | |
| 1^st^ quartile | 5842 (23.7) | 2630 (45.0) | Reference | Reference | 1138 (19.5) | Reference | Reference |
| 2^nd^ | 6098 (24.7) | 2348 (38.5) | **0.86 (0.82, 0.89)** | 0.96 (0.93, 1.00) | 1021 (16.7) | **0.86 (0.80, 0.93)** | 1.00 (0.92, 1.07) |
| 3^rd^ | 6084 (24.7) | 1817 (29.9) | **0.66 (0.63, 0.70)** | **0.79 (0.75, 0.82**) | 838 (13.8) | **0.71 (0.65, 0.77)** | **0.86 (0.79, 0.93)** |
| 4^th^ | 3243 (13.2) | 714 (22.0) | **0.49 (0.46, 0.52)** | **0.58 (0.54, 0.62)** | 389 (12.0) | **0.62 (0.55, 0.69)** | **0.76 (0.68, 0.85)** |
| Missing | 3402 (13.8) | 996 (29.3) | **0.65 (0.61, 0.69)** | **0.84 (0.78, 0.90)** | 478 (14.1) | **0.72 (0.65, 0.80)** | **0.84 (0.75, 0.95)** |
| **Site** | | | | | | | |
| Alabama | 266 (1.1) | 80 (30.1) | **1.35 (1.11, 1.63)** | **1.44 (1.18, 1.77)** | 62 (23.3) | **2.12 (1.68, 2.67)** | **2.72 (2.08, 3.56)** |
| Arkansas | 2161 (8.8) | 637 (29.5) | **1.32 (1.21, 1.43)** | **1.31 (1.20, 1.42)** | 170 (7.9) | **0.71 (0.61, 0.84)** | **0.65 (0.55, 0.76)** |
| Arizona | 3373 (13.7) | 928 (27.5) | **1.23 (1.14, 1.33)** | **1.25 (1.16, 1.35)** | 316 (9.4) | **0.85 (0.75, 0.97)** | 0.89 (0.78, 1.01) |
| Colorado | 1663 (6.7) | 746 (44.9) | **2.01 (1.86, 2.16)** | **1.92 (1.78, 2.08)** | 414 (24.9) | **2.26 (2.02, 2.54)** | **1.80 (1.6, 2.03)** |
| Georgia | 4944 (20.0) | 1104 (22.3) | Reference | Reference | 544 (11.0) | Reference | Reference |
| Maryland | 1023 (4.2) | 544 (53.2) | **2.38 (2.20, 2.57)** | **2.27 (2.10, 2.46)** | 238 (23.3) | **2.11 (1.84, 2.42)** | **1.85 (1.61, 2.13)** |
| Minnesota | 546 (2.2) | 213 (39.0) | **1.75 (1.55, 1.96)** | **1.72 (1.52, 1.94)** | 77 (14.1) | **1.28 (1.03, 1.60)** | 1.02 (0.81, 1.28) |
| North Carolina | 3389 (13.7) | 454 (13.4) | **0.60 (0.54, 0.66)** | **0.60 (0.54, 0.66)** | 226 (6.7) | **0.61 (0.52, 0.70)** | **0.58 (0.50, 0.68)** |
| New Jersey | 3644 (14.8) | 2380 (65.3) | **2.93 (2.76, 3.10)** | **2.79 (2.63, 2.97)** | 1002 (27.5) | **2.50 (2.27, 2.75)** | **2.08 (1.89, 2.30)** |
| South Carolina | 1175 (4.8) | 294 (25.0) | 1.12 (1.00, 1.25) | **1.16 (1.03, 1.30)** | 164 (14.0) | **1.27 (1.08, 1.49)** | **1.43 (1.21, 1.69)** |
| Tennessee | 792 (3.2) | 264 (33.3) | **1.49 (1.34, 1.67)** | **1.45 (1.29, 1.62)** | 208 (26.3) | **2.39 (2.07, 2.75)** | **1.88 (1.62, 2.19)** |
| Utah | 1114 (4.5) | 546 (49.0) | **2.19 (2.03, 2.38)** | **2.14 (1.97, 2.33)** | 238 (21.4) | **1.94 (1.69, 2.23)** | **1.84 (1.59, 2.12)** |
| Wisconsin | 579 (2.4) | 315 (54.4) | **2.44 (2.22, 2.67)** | **2.58 (2.34, 2.85)** | 205 (35.4) | **3.22 (2.81, 3.69)** | **2.64 (2.27, 3.07)** |
| **Study Year** | | | | | | | |
| 2000 | 735 (3.0) | 173 (23.5) | Reference | Reference | 63 (8.6) | Reference | Reference |
| 2002 | 1402 (5.7) | 365 (26.0) | 1.11 (0.95, 1.29) | 0.97 (0.83, 1.14) | 124 (8.8) | 1.03 (0.77, 1.38) | 1.02 (0.76, 1.36) |
| 2004 | 882 (3.6) | 166 (18.8) | **0.80 (0.66, 0.97)** | 0.91 (0.75, 1.10) | 65 (7.4) | 0.86 (0.62, 1.20) | 0.85 (0.61, 1.18) |
| 2006 | 1670 (6.8) | 397 (23.8) | 1.01 (0.86, 1.18) | 1.03 (0.88, 1.21) | 180 (10.8) | 1.26 (0.96, 1.65) | 1.10 (0.83, 1.46) |
| 2008 | 2139 (8.7) | 478 (22.4) | 0.95 (0.82, 1.11) | 0.99 (0.85, 1.15) | 158 (7.4) | 0.86 (0.65, 1.14) | 0.90 (0.68, 1.19) |
| 2010 | 4137 (16.8) | 1459 (35.3) | **1.50 (1.31, 1.72)** | 1.09 (0.94, 1.25) | 535 (12.9) | **1.51 (1.18, 1.94)** | 1.29 (1.00, 1.67) |
| 2012 | 4189 (17.0) | 1690 (40.3) | **1.71 (1.50, 1.96)** | **1.20 (1.04, 1.38)** | 833 (19.9) | **2.32 (1.82, 2.96)** | **1.79 (1.39, 2.30)** |
| 2014 | 4620 (18.7) | 1908 (41.3) | **1.75 (1.53, 2.01)** | **1.22 (1.06, 1.40)** | 908 (19.7) | **2.29 (1.80, 2.92)** | **1.77 (1.38, 2.29)** |
| 2016 | 4895 (19.8) | 1869 (38.2) | **1.62 (1.42, 1.86)** | 1.02 (0.88, 1.17) | 998 (20.4) | **2.38 (1.87, 3.03)** | **1.64 (1.27, 2.12)** |

^a^Bolded estimate indicates the confidence interval excludes one

^b^Adjusted for site and study year; site controlled only for study year; study year controlled only for site

IQ: intelligence quotient; NH: non-Hispanic; ASD: autism spectrum disorder; yrs: years; DSM-IV-TR: Diagnostic and Statistical Manual of Mental Disorders, Fourth Edition, Text Revision.

Supplementary Table 2. Variables included in the multiple imputation model

| **Order of Imputation** | **Description** | **Abbreviation for Supplementary Figures 1-4** | **% Missing** | **Spearman Correlation** | | | | **Imputation Method** |
| --- | --- | --- | --- | --- | --- | --- | --- | --- |
|  |  |  |  | **Any Adaptive Score** | | **IQ** | |  |
|  |  |  |  | **Score** | **Recorded** | **Score** | **Recorded** |  |
| NA | Study year | NA | 0.00% | 0.19 | -0.12 | 0.08 | -0.13 | NA |
| NA | Study site | NA | 0.00% | NA | NA | NA | NA | NA |
| NA | Child’s sex | NA | 0.00% | 0.04 | -0.01 | 0.07 | 0.01 | NA |
| NA | Age at earliest evaluation abstracted | NA | 0.00% | 0.10 | -0.19 | 0.23 | -0.05 | Bayesian linear regression |
| 1 | General delay before age 3 coded on any evaluation | General delay | 0.01% | -0.10 | 0.17 | -0.18 | 0.06 | Logistic regression |
| 2 | Social delay before age 3 coded on any evaluation | Social delay | 0.01% | -0.08 | 0.17 | -0.17 | 0.06 | Logistic regression |
| 3 | Language delay before age 3 coded on any evaluation | Language delay | 0.01% | -0.13 | 0.18 | -0.24 | 0.06 | Logistic regression |
| 4 | Developmental plateau or regression coded on any evaluation | Development reg | 0.01% | -0.14 | 0.08 | -0.18 | 0.00 | Logistic regression |
| 5 | Age at earliest known ASD diagnosis | Age at diagnosis | 0.19% | 0.11 | -0.08 | 0.16 | 0.05 | Polytomous logistic regression |
| 6 | Had at least one school source in their abstracted records | School source | 0.46% | -0.04 | 0.25 | -0.04 | 0.33 | Logistic regression |
| 7 | Child’s race and ethnicity | Child’s race | 1.85% | NA | NA | NA | NA | Polytomous logistic regression |
| 8 | Abnormalities in the development of cognitive skills indicated in records | Abn cog develop | 3.88% | 0.21 | 0.26 | 0.18 | 0.38 | Logistic regression |
| 9 | Hyperactivity or attention deficits indicated in records | Hyperactivity/att def | 3.88% | 0.03 | 0.08 | 0.03 | 0.15 | Logistic regression |
| 10 | Lack of fear or excessive fearfulness indicated in records | Lack/excess fear | 3.88% | -0.07 | 0.16 | -0.07 | 0.08 | Logistic regression |
| 11 | Abnormalities in mood or affect indicated in records | Abn mood/affect | 3.88% | 0.10 | 0.06 | 0.13 | 0.11 | Logistic regression |
| 12 | Number of associated features indicated in records | Total features | 3.88% | -0.04 | 0.23 | 0.00 | 0.22 | Logistic regression |
| 13 | At least one externalizing associated feature indicated records | 1+ Externalizing features | 3.88% | 0.02 | 0.06 | 0.02 | 0.10 | Logistic regression |
| 14 | Clinician reviewer’s estimate of child’s level of ASD-associated impairment | Clin-rated imp lev | 3.90% | -0.30 | 0.09 | -0.31 | -0.05 | Logistic regression |
| 15 | Percent of families with children <18 living in poverty (tertiles) | % fam in poverty | 6.71% | 0.12 | -0.04 | 0.19 | 0.01 | Polytomous logistic regression |
| 16 | Median household income (tertiles) | Median income | 6.75% | 0.14 | -0.04 | 0.21 | 0.00 | Polytomous logistic regression |
| 17 | DSM-IV-TR 1c criterion coded on any evaluation | DSM-IV-TR 1c coded | 13.80% | -0.07 | 0.17 | -0.14 | 0.07 | Logistic regression |
| 18 | DSM-IV-TR 2a criterion coded on any evaluation | DSM-IV-TR 2a coded | 13.80% | -0.16 | 0.17 | -0.28 | 0.08 | Logistic regression |
| 29 | DSM-IV-TR 2b criterion coded on any evaluation | DSM-IV-TR 2b coded | 13.80% | 0.09 | 0.12 | 0.02 | 0.16 | Logistic regression |
| 20 | DSM-IV-TR 2d criterion coded on any evaluation | DSM-IV-TR 2d coded | 13.80% | -0.16 | 0.20 | -0.21 | 0.07 | Logistic regression |
| 21 | DSM-IV-TR 3d criterion coded on any evaluation | DSM-IV-TR 3d coded | 13.80% | -0.06 | 0.15 | -0.07 | 0.08 | Logistic regression |
| 22 | Number of DSM-IV-TR social and communication criteria coded on any evaluation | DSM soc com total | 13.80% | -0.07 | 0.27 | -0.16 | 0.16 | Predictive mean matching |
| 23 | Number of DSM-IV-TR restricted and repetitive behaviors and/or interests criteria coded on any evaluation | DSM rep/restr tot | 13.80% | -0.01 | 0.17 | -0.02 | 0.12 | Predictive mean matching |
| 24 | Number of restricted and repetitive behaviors and/or interests discriminators in records | Rep/restr disc | 13.79% | 0.03 | 0.10 | 0.03 | 0.08 | Predictive mean matching |
| 25 | Number of social and communication discriminators in records | Soc com disc | 13.79% | -0.18 | 0.17 | -0.19 | 0.05 | Predictive mean matching |
| 26 | Maternal education | Maternal educ | 34.48% | 0.19 | -0.01 | 0.20 | 0.01 | Polytomous logistic regression |
| 27 | IQ test score | IQ | 15.66% | 0.56 | -0.25 | NA | NA | Bayesian linear regression |
| 28 | Non-VABS test score or score from a test administered before age 6 | Other adapt score | 41.12% | 0.93 | NA | 0.54 | 0.10 | Bayesian linear regression |
| 29 | Score from a VABS test administered between 6-8 years of age | VABS 6-8yrs score | 83.34% | 0.98 | NA | 0.63 | 0.11 | Bayesian linear regression |

IQ: intelligence quotient; NA: not applicable; ASD: autism spectrum disorder; DSM-IV-TR: Diagnostic and Statistical Manual or Mental Disorders, Fourth Edition, Text Revision; VABS: Vineland Adaptive Behavior Scales.

Supplementary Table 3. Conversion of categorical intellectual and adaptive classifications to standard scores prior to imputation

| **Description of Test Result Recorded** | **Conversion Method** | **Adaptive Score**  **n (%)** | **IQ Score**  **n (%)** |
| --- | --- | --- | --- |
| ># or <# | Score set to # | 57 (0.2) | 492 (2.0) |
| Non-standard score or categorical test result recorded |  |  |  |
| a) Functioning level corresponding to specific score range | a) Score set to midpoint of range | 543 (2.2) | 2579 (10.5) |
| b) “Significant Adaptive Impairment – NOS” or  “Intellectual Disability, NOS” | b) Score set to 63 (midpoint between 1 and 2 standard deviations below the mean) | 41 (0.2) | 244 (1.0) |
| c) “No Significant Adaptive Impairment – NOS” or “Above ID range, NOS” | c) Score set to 100 (average) | 19 (0.1) | 84 (0.3) |
| d) Above average functioning (score >115) | d) Score set to 130 (2 standard deviations above the mean) | <10 (0.0) | 61 (0.2) |
| Score outside of plausible range for that adaptive test | Score set to minimum or maximum for that adaptive test, as appropriate | 48 (0.2) | NA |

IQ: intelligence quotient; ID: intellectual disability; NOS: not otherwise specified; NA: not applicable.

Supplementary Table 4. Additional details about the multiple imputation procedure

| **Software** | R version 4.2.2 |
| --- | --- |
| **R package** | mice version 3.16.0 |
| ***Step 1*** | |
| **Number of imputations** | 1 |
| **Number of iterations** | 50 |
| **Seed number** | 1234 |
| ***Step 2*** | |
| **Number of imputations** | 35 |
| **Number of iterations** | 50 |
| **Seed number** | 1234 |

Supplementary Figure 1. Trace plots from the first step of the multiple imputation procedure during which adaptive test scores from non-Vineland Adaptive Behavior Scales (VABS) tests or obtained before age six were converted to VABS 6 to 8 years scores; see Supplementary Table 2 for variable descriptions (continued on next page)
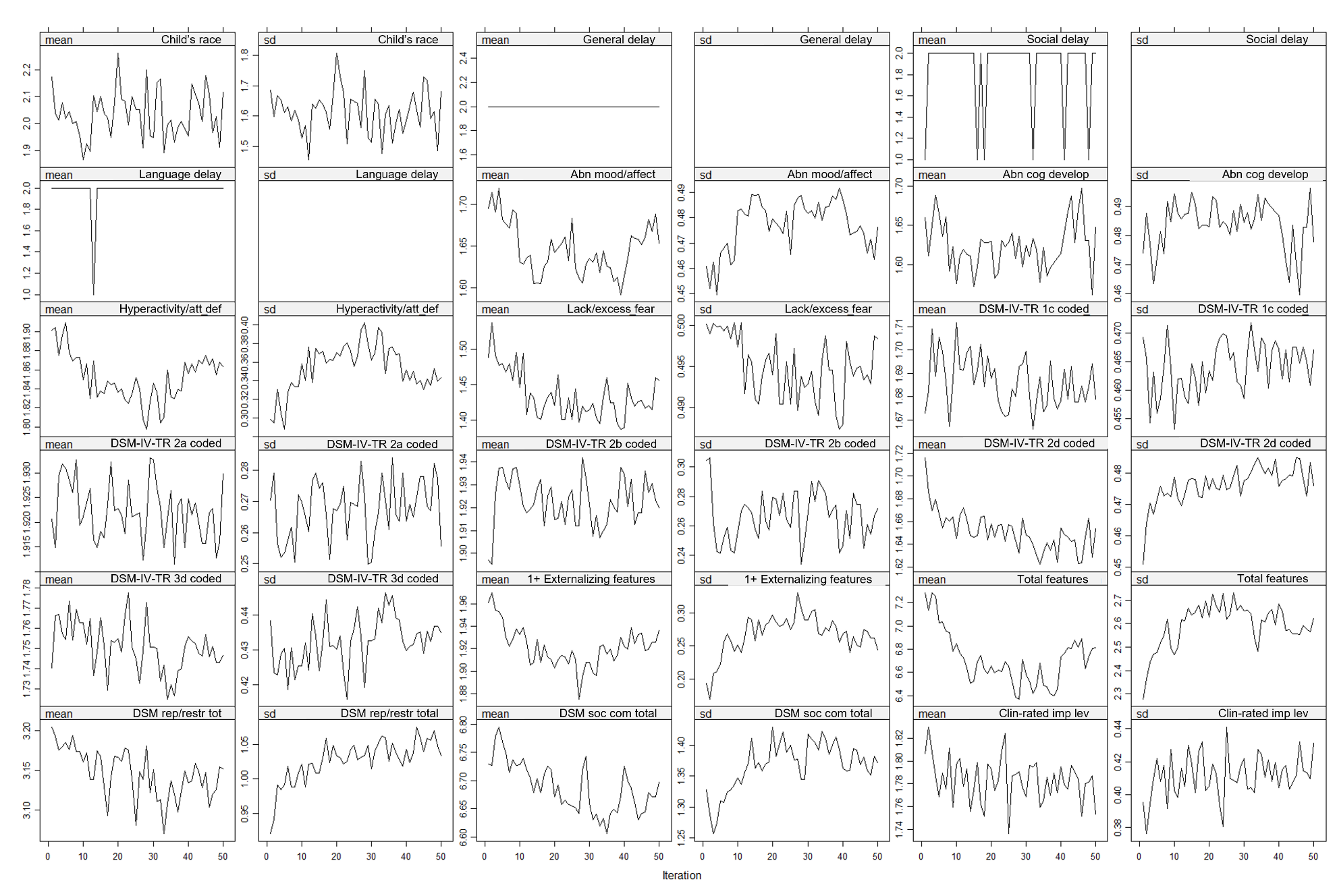


**Supplementary Figure 1 (continued).** Trace plots from the first step of the multiple imputation procedure during which adaptive test scores from non- Vineland Adaptive Behavior Scales (VABS) tests or obtained before age six were converted to VABS 6 to 8 years scores; see Supplementary Table 2 for variable descriptions


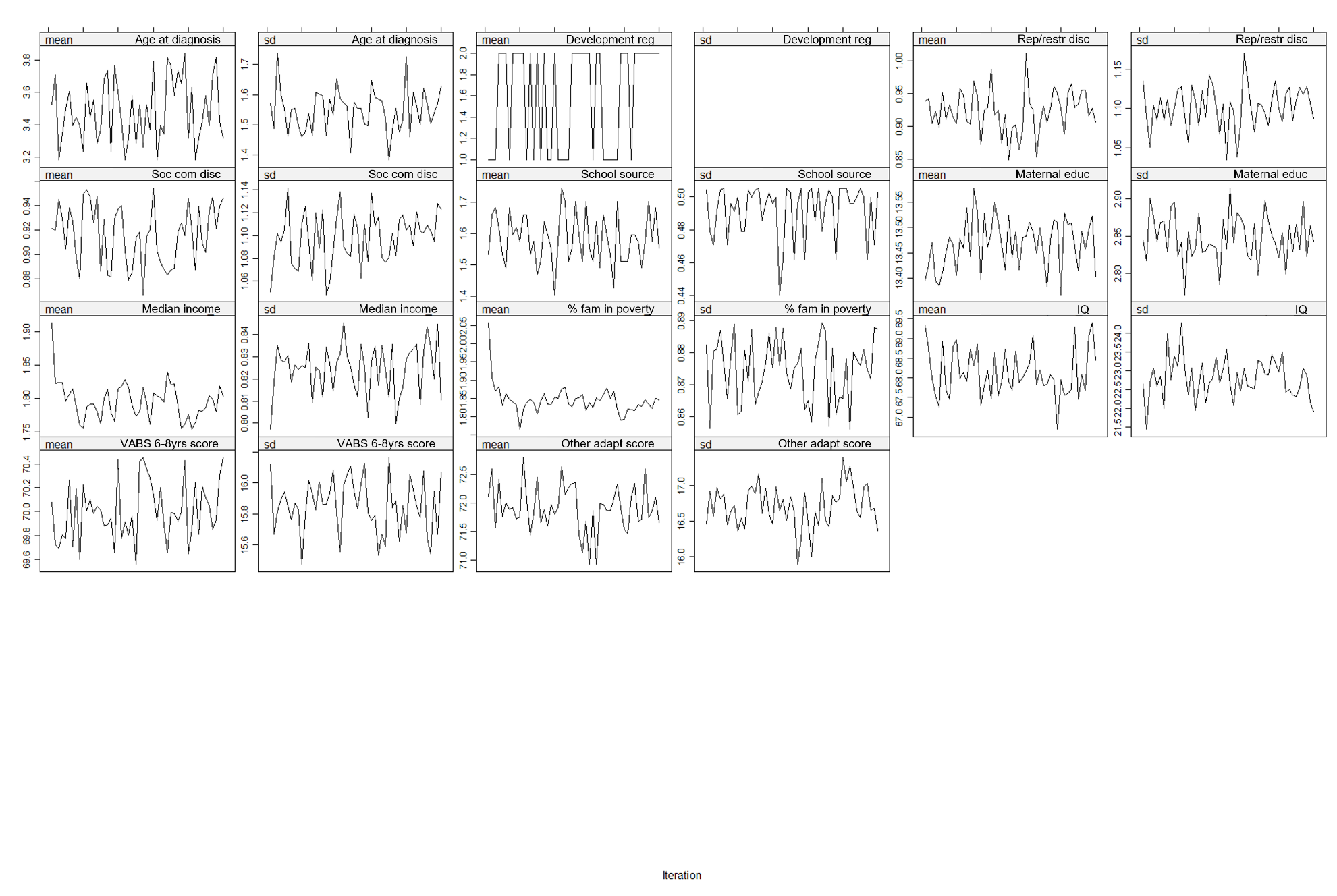


Supplementary Figure 2. Trace plots from the second step of the multiple imputation procedure during which Vineland Adaptive Behavior Scales 6 to 8 years scores and any other missing variables were imputed; see Supplementary Table 2 for variable descriptions (continued on next page)


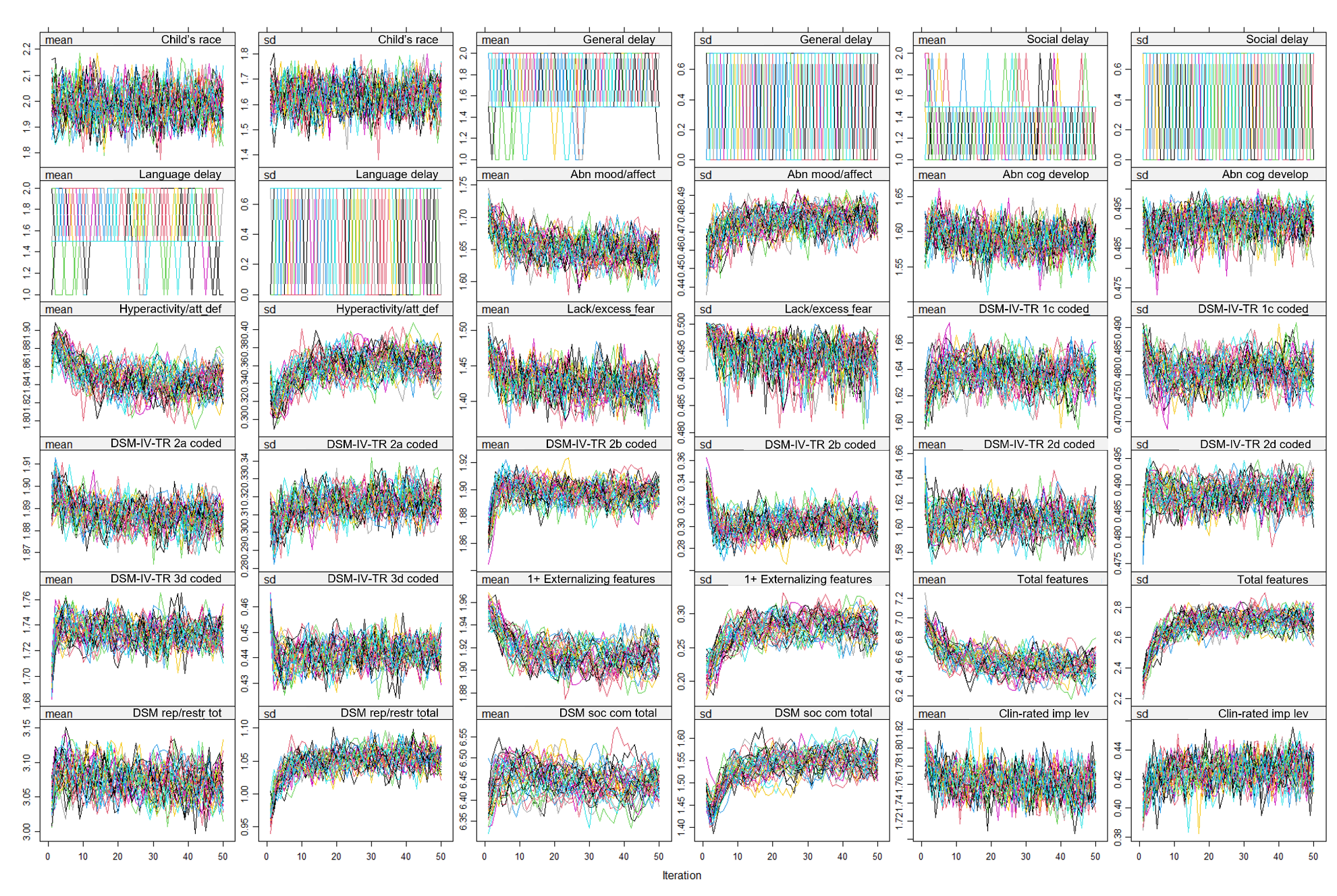


**Supplementary Figure 2 (continued).** Trace plots from the second step of the multiple imputation procedure during which Vineland Adaptive Behavior Scales 6 to 8 years scores and any other missing variables were imputed; see Supplementary Table 2 for variable descriptions


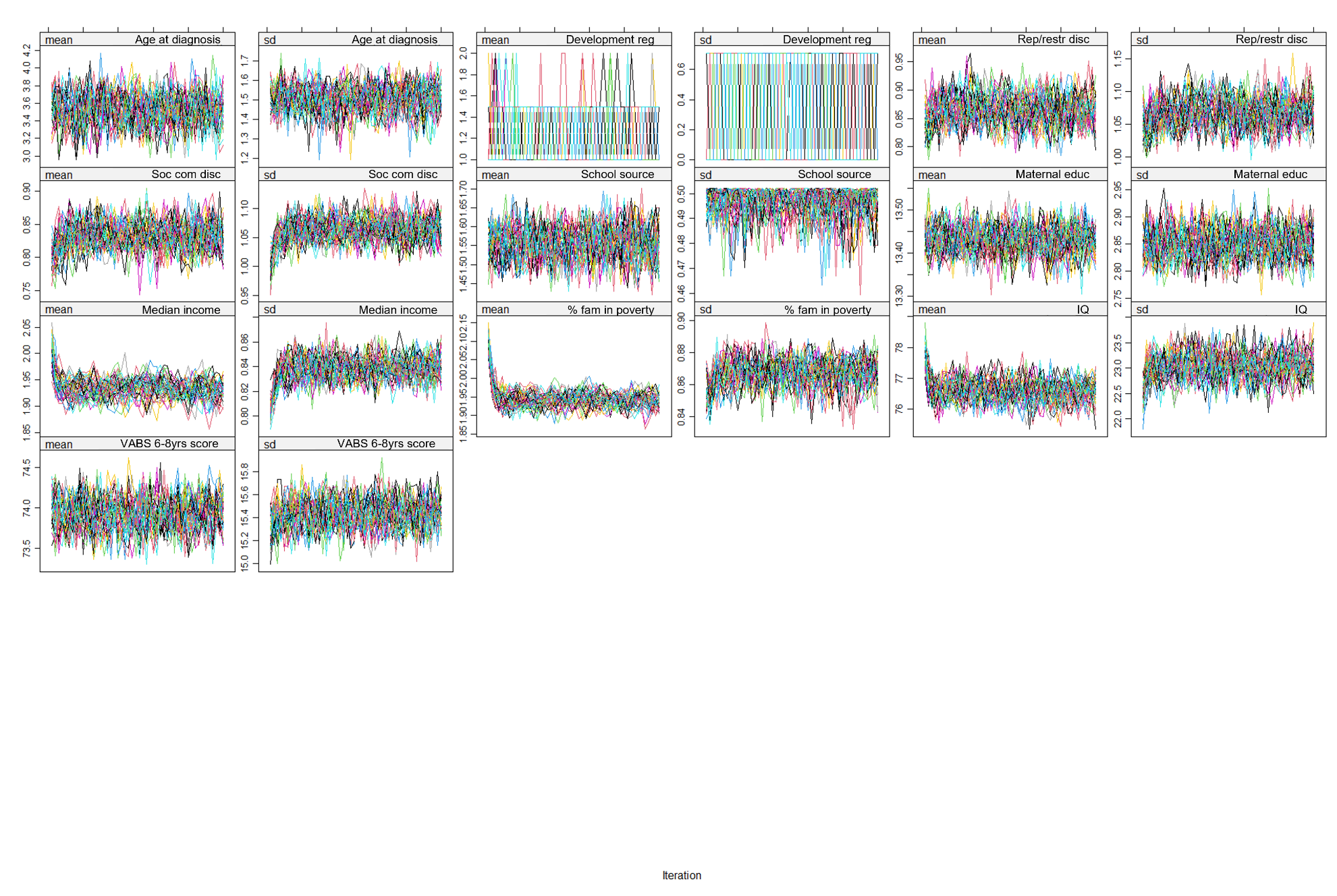


Supplementary Figure 3. Comparison of observed (imputation 0) and imputed (imputation 1-35) values for numeric variables; see Supplementary Table 2 for variable descriptions


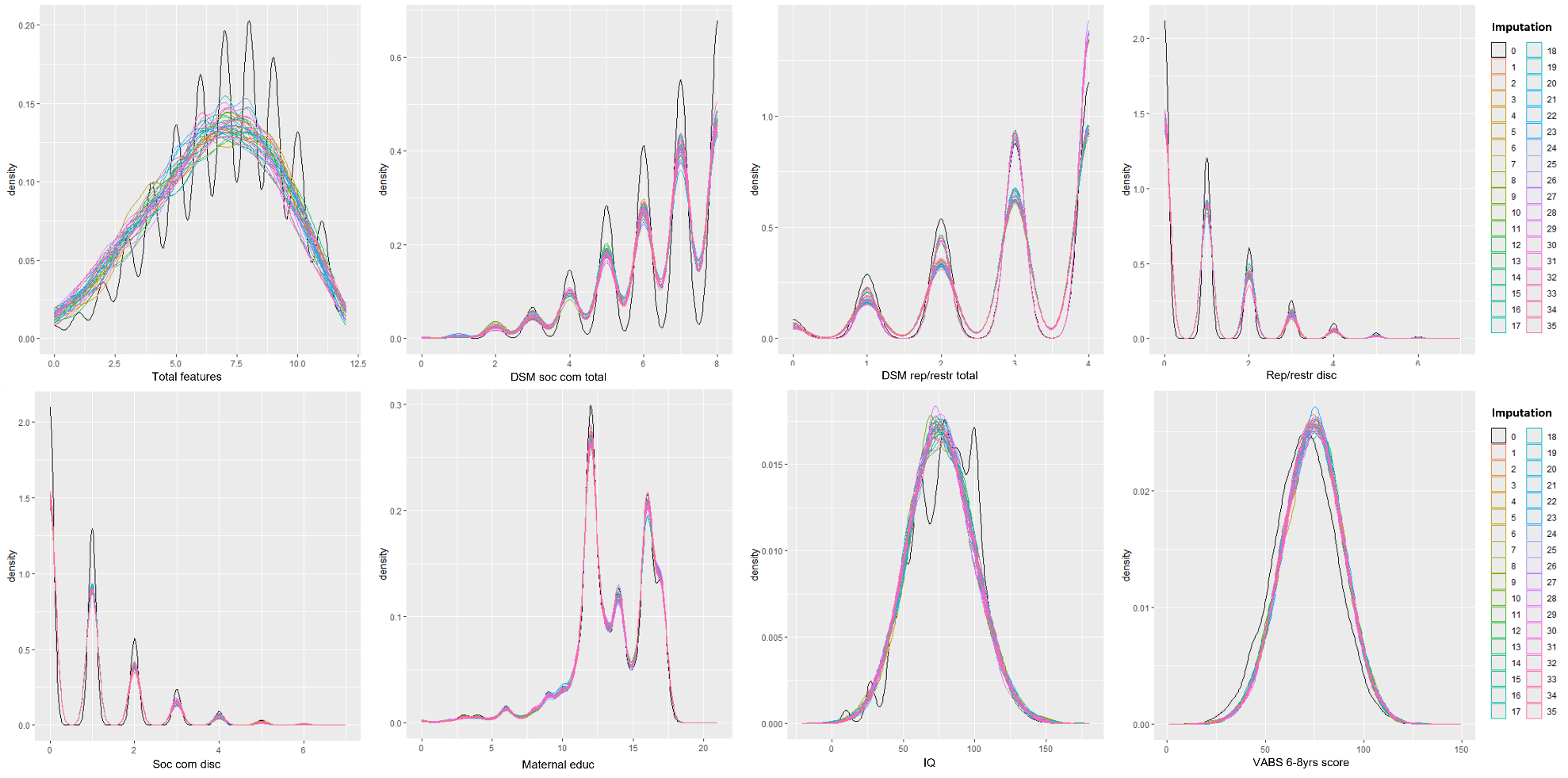


Supplementary Figure 4. Comparison of observed (imputation 0) and imputed (imputation 1-35) values^a^ for categorical variables; see Supplementary Table 2 for variable descriptions


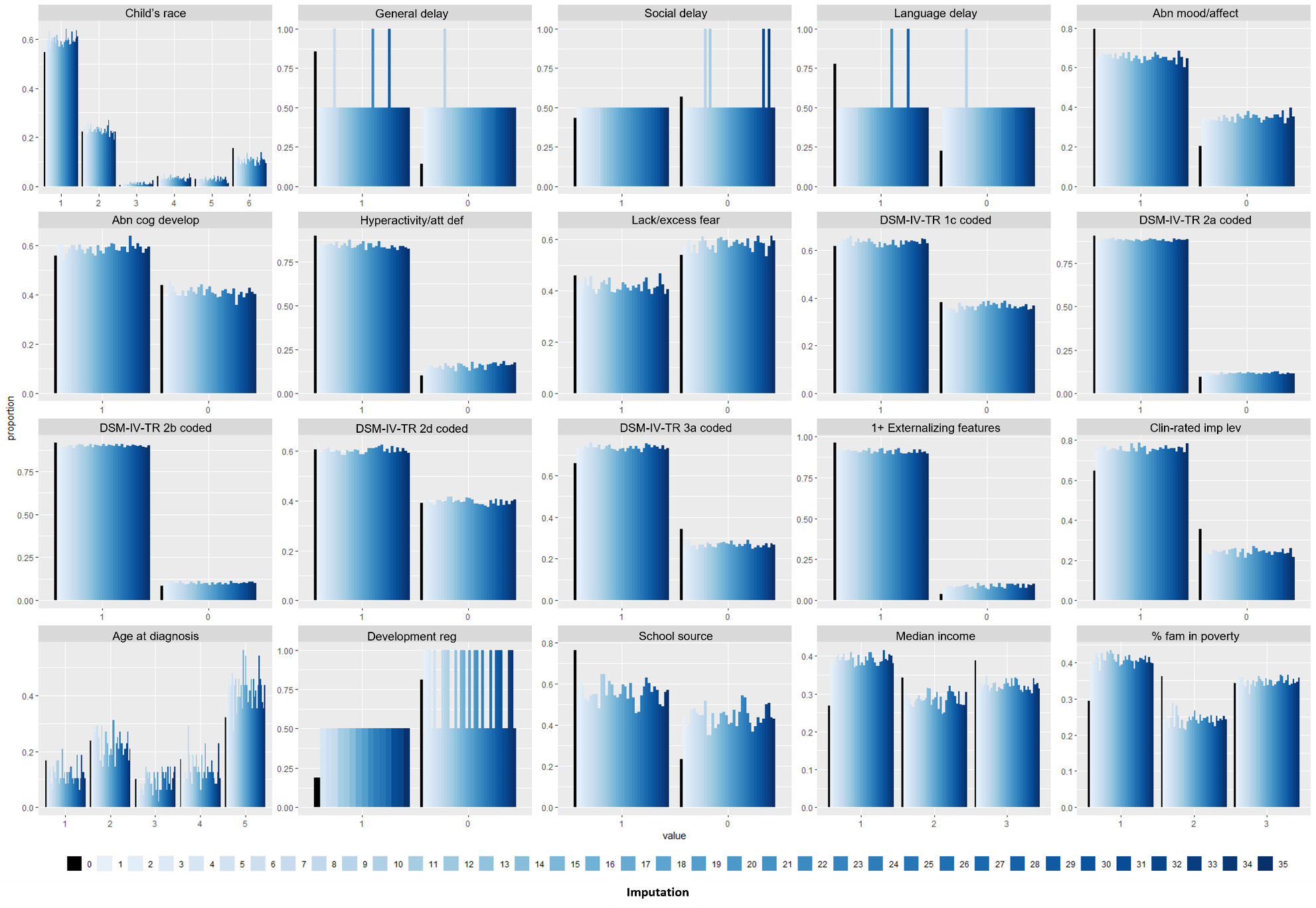


^a^Values: Child’s race – 1 non-Hispanic (NH) White, 2 NH Black, 3 NH American Indian or Alaska Native, 4 NH Asian or Pacific Islander, 5 NH other or multiracial, 6 Hispanic; General delay, social delay, language delay, abn mood/affect, abn cog develop, hyperactivity/att def, lack/excess fear, DSM-IV-TR coded, 1+ externalizing features, development reg, school source – 0 no, 1 yes; Clin-rated imp lev – 0 low, 1 high; Age at diagnosis – 1 0-2 years, 2 preschool/3-4 years, 3 kindergarten/5 years, 4 1^st^ or 2^nd^ grade/6+ years, 5 no documented diagnosis; Median income, % fam in poverty – 1 lowest tertile, 2 middle tertile, 3 highest tertile
