# Supplementary material for: Trends Over Time in the Prevalence of Autism by Adaptive and Intellectual Functioning Levels
Source: Autism Res. 2025 Dec 28;19(1):e70167. doi: 10.1002/aur.70167 (PMC12853238; doi:10.1002/aur.70167)
Supplement: Supplementary file 2 — Data S2: aur70167‐sup‐0002‐SupplementaryMaterials2.docx. [file AUR-19-0-s001.docx]

**Supplementary Analyses**

**Contents**

[Supplementary Analysis 1: Complete Case Analysis 2](#_Toc205385112)

[Supplementary Table 5. Prevalence of autism by adaptive level in 8-year-old children in the United States, Autism and Developmental Disabilities Monitoring Network, 2000-2016 3](#_Toc205385113)

[Supplementary Table 6. Prevalence of autism with and without co-occurring intellectual disability in 8-year-old children in the United States, Autism and Developmental Disabilities Monitoring Network, 2000-2016 4](#_Toc205385114)

[Supplementary Analysis 2: Multiply imputed data based on imputation of the most recent adaptive score regardless of which test or at what age the test was administered 7](#_Toc205385115)

[Supplementary Table 7. Prevalence of autism by adaptive level in 8-year-old children in the United States, Autism and Developmental Disabilities Monitoring Network, 2000-2016 7](#_Toc205385116)

[Supplementary Table 8. Prevalence of autism with and without co-occurring intellectual disability in 8-year-old children in the United States, Autism and Developmental Disabilities Monitoring Network, 2000-2016 8](#_Toc205385117)

[Supplementary Analysis 3: Multiply imputed data limited to sites that participated in at least eight of the nine study years (Arizona, Georgia, North Carolina) 10](#_Toc205385118)

[Supplementary Table 9. Prevalence of autism by adaptive level in 8-year-old children in the United States, Autism and Developmental Disabilities Monitoring Network, 2000-2016 10](#_Toc205385119)

[Supplementary Table 10. Prevalence of autism with and without co-occurring intellectual disability in 8-year-old children in the United States, Autism and Developmental Disabilities Monitoring Network, 2000-2016 12](#_Toc205385120)

# Supplementary Analysis 1: Complete Case Analysis

To produce prevalence estimates based on complete cases, we assumed that the proportion of children (a) within each adaptive level and (b) with and without co-occurring intellectual disability was the same in complete cases and in total cases. We multiplied the total case number for each study year by the percentage of complete cases within each adaptive or intellectual disability category in the same year, obtaining an estimate of the total cases in each category, which was used to calculate prevalence.

Supplementary Table 5. Prevalence of autism by adaptive level in 8-year-old children in the United States, overall and stratified by child’s sex, Autism and Developmental Disabilities Monitoring Network, 2000-2016; complete case analysis limited to children with any adaptive score recorded

| **Level of Adaptive Challenges** | **Study Year** | | | | | | | | |
| --- | --- | --- | --- | --- | --- | --- | --- | --- | --- |
|  | **2000** | **2002** | **2004** | **2006** | **2008** | **2010** | **2012** | **2014** | **2016** |
| **Overall** | | | | | | | | | |
| None | 0.4 (0.3-0.5) | 0.6 (0.5-0.7) | 0.8 (0.6-0.9) | 1.1 (0.9-1.3) | 2.1 (1.9-2.3) | 3.0 (2.8-3.2) | 3.1 (2.9-3.3) | 3.6 (3.4-3.8) | 3.6 (3.3-3.8) |
| Borderline | 1.2 (1.0-1.4) | 1.3 (1.1-1.4) | 1.5 (1.3-1.7) | 2.1 (1.9-2.3) | 3.8 (3.5-4.1) | 5.5 (5.3-5.8) | 5.4 (5.1-5.7) | 5.7 (5.4-6.0) | 6.8 (6.5-7.1) |
| Mild | 3.4 (3.1-3.8) | 3.7 (3.5-4.0) | 4.3 (3.9-4.7) | 5.0 (4.6-5.3) | 6.4 (6.0-6.8) | 7.1 (6.8-7.5) | 6.1 (5.8-6.4) | 6.9 (6.6-7.2) | 7.2 (6.9-7.5) |
| Moderate to Profound | 1.4 (1.2-1.7) | 1.2 (1.1-1.4) | 1.3 (1.1-1.5) | 1.4 (1.2-1.6) | 1.2 (1.1-1.4) | 1.5 (1.3-1.6) | 1.1 (1-1.2) | 1.2 (1.1-1.4) | 1.3 (1.2-1.5) |
| **Boys** | | | | | | | | | |
| None | 0.7 (0.5-1.0) | 1.0 (0.8-1.2) | 1.3 (1.0-1.6) | 1.6 (1.4-1.9) | 3.5 (3.1-4.0) | 4.9 (4.6-5.3) | 5.1 (4.7-5.5) | 5.6 (5.2-6.0) | 5.7 (5.3-6.1) |
| Borderline | 1.9 (1.6-2.3) | 2.2 (1.9-2.5) | 2.5 (2.1-2.9) | 3.5 (3.1-3.9) | 6.4 (5.9-7.0) | 9.1 (8.6-9.6) | 8.6 (8.2-9.1) | 9.3 (8.8-9.8) | 11.1 (10.5-11.7) |
| Mild | 5.5 (4.9-6.2) | 6.1 (5.6-6.5) | 7.0 (6.3-7.7) | 8.0 (7.4-8.6) | 10.3 (9.6-11.0) | 11.5 (10.9-12.1) | 9.8 (9.3-10.3) | 10.8 (10.3-11.4) | 11.5 (10.9-12.1) |
| Moderate to Profound | 1.9 (1.6-2.3) | 1.8 (1.5-2.0) | 1.9 (1.6-2.3) | 2.1 (1.8-2.4) | 1.9 (1.6-2.2) | 2.2 (2.0-2.5) | 1.8 (1.6-2.0) | 2.0 (1.7-2.2) | 2.0 (1.7-2.2) |
| **Girls** | | | | | | | | | |
| None | 0.1 (0.0-0.2) | 0.2 (0.1-0.3) | 0.2 (0.1-0.4) | 0.5 (0.4-0.7) | 0.6 (0.5-0.8) | 0.9 (0.8-1.1) | 1.0 (0.8-1.2) | 1.4 (1.2-1.7) | 1.3 (1.1-1.5) |
| Borderline | 0.5 (0.3-0.7) | 0.3 (0.2-0.5) | 0.5 (0.3-0.7) | 0.6 (0.5-0.8) | 1.1 (0.9-1.4) | 1.9 (1.7-2.2) | 2.0 (1.8-2.3) | 2.0 (1.8-2.2) | 2.3 (2.0-2.6) |
| Mild | 1.3 (1.0-1.7) | 1.3 (1.1-1.5) | 1.5 (1.2-1.9) | 1.7 (1.5-2.0) | 2.3 (2.0-2.6) | 2.6 (2.3-2.9) | 2.2 (2.0-2.5) | 2.8 (2.6-3.2) | 2.7 (2.5-3.1) |
| Moderate to Profound | 0.9 (0.7-1.2) | 0.6 (0.5-0.8) | 0.6 (0.4-0.8) | 0.7 (0.5-0.9) | 0.6 (0.4-0.8) | 0.7 (0.5-0.8) | 0.4 (0.3-0.6) | 0.5 (0.4-0.6) | 0.7 (0.5-0.8) |

Supplementary Table 6. Prevalence of autism with and without co-occurring intellectual disability (ID) in 8-year-old children in the United States, overall and stratified by child’s sex, Autism and Developmental Disabilities Monitoring Network, 2000-2016; complete case analysis limited to (A) children with any adaptive score recorded and (B) children with any adaptive score recorded and less than one year between their adaptive and IQ tests

| **Co-Occurring ID** | **Study Year** | | | | | | | | |
| --- | --- | --- | --- | --- | --- | --- | --- | --- | --- |
|  | **2000** | **2002** | **2004** | **2006** | **2008** | **2010** | **2012** | **2014** | **2016** |
| **(A)** **Limited to children with both an IQ and any adaptive test score recorded** | | | | | | | | | |
| **ADDM Surveillance Definition (IQ≤70)** | | | | | | | | | |
| **Overall** | | | | | | | | | |
| ID | 3.6 (3.2-3.9) | 3.6 (3.4-3.9) | 3.9 (3.5-4.2) | 4.5 (4.2-4.8) | 5.9 (5.5-6.3) | 6.6 (6.3-6.9) | 5.8 (5.5-6.1) | 6.1 (5.8-6.4) | 7.3 (7.0-7.6) |
| No ID | 2.9 (2.6-3.2) | 3.2 (2.9-3.4) | 3.9 (3.6-4.3) | 5.0 (4.7-5.3) | 7.7 (7.3-8.1) | 10.5 (10.1-10.9) | 9.8 (9.5-10.2) | 11.3 (10.9-11.8) | 11.5 (11.1-12) |
| **Boys** | | | | | | | | | |
| ID | 5.1 (4.6-5.8) | 5.5 (5.1-6.0) | 6.0 (5.4-6.7) | 7.0 (6.4-7.5) | 9.2 (8.6-9.9) | 10.3 (9.7-10.9) | 9.1 (8.6-9.6) | 9.3 (8.8-9.8) | 11.4 (10.8-11.9) |
| No ID | 4.9 (4.4-5.6) | 5.4 (5.0-5.9) | 6.6 (6.0-7.3) | 8.2 (7.6-8.8) | 12.9 (12.1-13.7) | 17.4 (16.7-18.2) | 16.2 (15.5-16.9) | 18.4 (17.7-19.1) | 18.9 (18.1-19.6) |
| **Girls** | | | | | | | | | |
| ID | 1.9 (1.6-2.3) | 1.6 (1.4-1.9) | 1.6 (1.3-2.0) | 1.9 (1.6-2.2) | 2.3 (2.0-2.7) | 2.8 (2.5-3.1) | 2.5 (2.2-2.7) | 2.7 (2.4-3.0) | 3.1 (2.8-3.4) |
| No ID | 0.8 (0.6-1.1) | 0.8 (0.6-1.0) | 1.2 (0.9-1.5) | 1.6 (1.4-1.9) | 2.3 (2.0-2.6) | 3.3 (3.0-3.6) | 3.2 (2.9-3.6) | 4.0 (3.7-4.4) | 3.9 (3.6-4.3) |
| **Clinical Definition (both IQ and VABS 6-8yrs Composite ≤70)** | | | | | | | | | |
| **Overall** | | | | | | | | | |
| ID | 3.3 (3.0-3.7) | 3.2 (3.0-3.4) | 3.5 (3.2-3.9) | 3.8 (3.5-4.1) | 4.7 (4.4-5.0) | 5.0 (4.8-5.3) | 4.3 (4.0-4.5) | 4.5 (4.3-4.8) | 5.1 (4.8-5.4) |
| No ID | 3.2 (2.8-3.5) | 3.6 (3.3-3.9) | 4.3 (3.9-4.7) | 5.7 (5.4-6.1) | 8.9 (8.4-9.3) | 12.0 (11.6-12.5) | 11.4 (11.0-11.8) | 12.9 (12.5-13.4) | 13.7 (13.3-14.2) |
| **Boys** | | | | | | | | | |
| ID | 4.8 (4.2-5.4) | 4.9 (4.5-5.3) | 5.5 (4.9-6.1) | 5.9 (5.4-6.4) | 7.4 (6.8-8.0) | 7.9 (7.4-8.4) | 6.7 (6.3-7.2) | 6.8 (6.4-7.3) | 7.8 (7.3-8.3) |
| No ID | 5.3 (4.7-6.0) | 6.1 (5.6-6.6) | 7.2 (6.5-7.9) | 9.3 (8.7-9.9) | 14.7 (13.9-15.6) | 19.8 (19.0-20.6) | 18.5 (17.8-19.3) | 20.9 (20.2-21.7) | 22.4 (21.6-23.2) |
| **Girls** | | | | | | | | | |
| ID | 1.8 (1.4-2.2) | 1.4 (1.2-1.7) | 1.5 (1.2-1.9) | 1.6 (1.4-1.9) | 1.9 (1.6-2.2) | 2.1 (1.8-2.3) | 1.7 (1.5-2.0) | 2.1 (1.9-2.4) | 2.3 (2.0-2.6) |
| No ID | 1.0 (0.7-1.3) | 1.0 (0.8-1.2) | 1.3 (1.0-1.7) | 1.9 (1.6-2.2) | 2.7 (2.4-3.1) | 4.0 (3.7-4.4) | 4.0 (3.7-4.3) | 4.6 (4.3-5.0) | 4.7 (4.3-5.1) |
| **(B)** **Limited to children with both an IQ and any adaptive test score recorded and less than one year between the two tests** | | | | | | | | | |
| **ADDM Surveillance Definition (IQ≤70)** | | | | | | | | | |
| **Overall** | | | | | | | | | |
| ID | 4.0 (3.6-4.4) | 3.9 (3.7-4.2) | 4.1 (3.8-4.5) | 4.9 (4.6-5.2) | 6.4 (6.0-6.8) | 7.3 (7.0-7.7) | 6.1 (5.8-6.4) | 6.3 (6.0-6.6) | 8.0 (7.7-8.4) |
| No ID | 2.5 (2.2-2.8) | 2.9 (2.6-3.1) | 3.7 (3.3-4.1) | 4.6 (4.3-5.0) | 7.1 (6.7-7.6) | 9.8 (9.4-10.2) | 9.6 (9.2-10.0) | 11.1 (10.7-11.5) | 10.8 (10.4-11.2) |
| **Boys** | | | | | | | | | |
| ID | 5.7 (5.1-6.4) | 6.0 (5.6-6.5) | 6.4 (5.8-7.1) | 7.6 (7.0-8.2) | 10.3 (9.6-11.0) | 11.5 (10.9-12.1) | 9.5 (9.0-10.0) | 9.6 (9.1-10.1) | 12.5 (12.0-13.2) |
| No ID | 4.4 (3.9-5.0) | 4.9 (4.5-5.4) | 6.2 (5.6-6.9) | 7.6 (7.1-8.2) | 11.8 (11.1-12.6) | 16.2 (15.5-17.0) | 15.8 (15.1-16.5) | 18.1 (17.4-18.9) | 17.7 (17.0-18.4) |
| **Girls** | | | | | | | | | |
| ID | 2.1 (1.8-2.5) | 1.7 (1.5-2.0) | 1.7 (1.4-2.1) | 2.1 (1.8-2.4) | 2.3 (2.0-2.7) | 3.1 (2.7-3.4) | 2.6 (2.3-2.9) | 2.9 (2.6-3.2) | 3.3 (3.0-3.7) |
| No ID | 0.6 (0.5-0.9) | 0.7 (0.6-0.9) | 1.1 (0.9-1.4) | 1.5 (1.2-1.8) | 2.2 (1.9-2.6) | 3.0 (2.7-3.4) | 3.1 (2.8-3.5) | 3.8 (3.5-4.2) | 3.7 (3.3-4.0) |
| **Clinical Definition (both IQ and VABS 6-8yrs Composite ≤70)** | | | | | | | | | |
| **Overall** | | | | | | | | | |
| ID | 3.6 (3.3-4.0) | 3.5 (3.2-3.8) | 3.7 (3.4-4.1) | 4.1 (3.8-4.4) | 5.1 (4.8-5.5) | 5.5 (5.2-5.8) | 4.4 (4.1-4.6) | 4.6 (4.4-4.9) | 5.6 (5.3-5.8) |
| No ID | 2.8 (2.5-3.2) | 3.3 (3.0-3.5) | 4.1 (3.7-4.5) | 5.4 (5.1-5.8) | 8.5 (8.0-8.9) | 11.6 (11.1-12.0) | 11.3 (10.9-11.7) | 12.8 (12.4-13.2) | 13.3 (12.9-13.7) |
| **Boys** | | | | | | | | | |
| ID | 5.2 (4.6-5.8) | 5.4 (5.0-5.9) | 5.8 (5.2-6.5) | 6.4 (5.8-6.9) | 8.2 (7.6-8.8) | 8.8 (8.3-9.3) | 6.8 (6.4-7.3) | 6.9 (6.4-7.3) | 8.6 (8.1-9.1) |
| No ID | 4.9 (4.3-5.5) | 5.6 (5.1-6.0) | 6.8 (6.2-7.5) | 8.8 (8.2-9.5) | 13.9 (13.1-14.8) | 18.9 (18.2-19.7) | 18.4 (17.7-19.2) | 20.9 (20.1-21.6) | 21.6 (20.9-22.4) |
| **Girls** | | | | | | | | | |
| ID | 1.9 (1.6-2.3) | 1.5 (1.3-1.7) | 1.5 (1.2-1.9) | 1.7 (1.4-2.0) | 1.9 (1.6-2.2) | 2.2 (1.9-2.5) | 1.8 (1.6-2.0) | 2.3 (2.0-2.6) | 2.4 (2.2-2.7) |
| No ID | 0.8 (0.6-1.1) | 0.9 (0.8-1.1) | 1.3 (1.0-1.6) | 1.8 (1.6-2.1) | 2.7 (2.4-3.1) | 3.9 (3.6-4.3) | 3.9 (3.6-4.3) | 4.5 (4.1-4.8) | 4.6 (4.2-5.0) |

ID: intellectual disability

# Supplementary Analysis 2: Multiply imputed data based on imputation of the most recent adaptive score regardless of which test or at what age the test was administered

Supplementary Table 7. Prevalence of autism by adaptive level in 8-year-old children in the United States, overall and stratified by child’s sex, Autism and Developmental Disabilities Monitoring Network, 2000-2016; multiply imputed data based on imputation of the most recent adaptive score regardless of which test or at what age the test was administered

| **Level of Adaptive Challenges** | **Study Year** | | | | | | | | |
| --- | --- | --- | --- | --- | --- | --- | --- | --- | --- |
|  | **2000** | **2002** | **2004** | **2006** | **2008** | **2010** | **2012** | **2014** | **2016** |
| **Overall** | | | | | | | | | |
| None | 0.6 (0.4-0.7) | 0.7 (0.6-0.9) | 0.9 (0.7-1.1) | 1.2 (1.1-1.4) | 2.3 (2.1-2.6) | 3.5 (3.2-3.7) | 3.5 (3.3-3.8) | 4.0 (3.7-4.3) | 4.0 (3.8-4.3) |
| Borderline | 1.4 (1.2-1.7) | 1.5 (1.3-1.7) | 1.7 (1.4-2.0) | 2.3 (2.1-2.6) | 4.0 (3.7-4.4) | 5.8 (5.5-6.1) | 5.5 (5.2-5.8) | 6.0 (5.6-6.3) | 6.8 (6.4-7.1) |
| Mild | 3.3 (2.9-3.6) | 3.5 (3.2-3.8) | 4.1 (3.7-4.5) | 4.7 (4.3-5.0) | 6.0 (5.6-6.4) | 6.6 (6.2-6.9) | 5.7 (5.3-6.0) | 6.4 (6.1-6.7) | 6.9 (6.5-7.2) |
| Moderate to Profound | 1.3 (1.1-1.5) | 1.1 (0.9-1.2) | 1.2 (1.0-1.4) | 1.3 (1.1-1.5) | 1.2 (1.0-1.4) | 1.3 (1.1-1.4) | 1.0 (0.8-1.1) | 1.1 (1.0-1.3) | 1.2 (1.1-1.4) |
| **Boys** | | | | | | | | | |
| None | 1.0 (0.7-1.3) | 1.2 (1.0-1.5) | 1.5 (1.2-1.9) | 1.9 (1.6-2.3) | 3.9 (3.5-4.4) | 5.8 (5.3-6.3) | 5.8 (5.4-6.3) | 6.4 (5.8-6.9) | 6.5 (6.0-7.0) |
| Borderline | 2.3 (1.9-2.7) | 2.6 (2.2-2.9) | 2.8 (2.4-3.3) | 3.9 (3.4-4.3) | 6.8 (6.2-7.4) | 9.5 (8.9-10.1) | 8.9 (8.4-9.5) | 9.6 (9.1-10.2) | 11.0 (10.4-11.6) |
| Mild | 5.2 (4.5-5.8) | 5.7 (5.2-6.1) | 6.6 (5.9-7.3) | 7.5 (6.9-8.1) | 9.7 (9.0-10.4) | 10.6 (10-11.2) | 9.1 (8.5-9.6) | 10 (9.5-10.6) | 10.9 (10.3-11.5) |
| Moderate to Profound | 1.8 (1.4-2.1) | 1.6 (1.3-1.8) | 1.8 (1.4-2.2) | 2.0 (1.6-2.3) | 1.8 (1.5-2.1) | 1.9 (1.7-2.2) | 1.6 (1.3-1.8) | 1.8 (1.5-2.0) | 1.8 (1.6-2.1) |
| **Girls** | | | | | | | | | |
| None | 0.2 (0.0-0.3) | 0.3 (0.1-0.4) | 0.3 (0.1-0.4) | 0.5 (0.4-0.7) | 0.7 (0.5-0.9) | 1.1 (0.9-1.3) | 1.2 (1.0-1.4) | 1.5 (1.3-1.8) | 1.5 (1.2-1.7) |
| Borderline | 0.5 (0.3-0.8) | 0.5 (0.3-0.6) | 0.6 (0.3-0.8) | 0.8 (0.6-1.0) | 1.2 (1.0-1.5) | 2.0 (1.7-2.3) | 2.1 (1.8-2.3) | 2.2 (1.9-2.5) | 2.4 (2.1-2.7) |
| Mild | 1.3 (1.0-1.7) | 1.2 (1.0-1.5) | 1.5 (1.2-1.9) | 1.7 (1.4-2.0) | 2.2 (1.8-2.5) | 2.4 (2.1-2.7) | 2.1 (1.9-2.4) | 2.7 (2.3-3.0) | 2.7 (2.3-3.0) |
| Moderate to Profound | 0.9 (0.6-1.1) | 0.6 (0.4-0.7) | 0.6 (0.4-0.8) | 0.6 (0.5-0.8) | 0.6 (0.4-0.7) | 0.6 (0.4-0.7) | 0.4 (0.3-0.5) | 0.5 (0.3-0.6) | 0.6 (0.4-0.7) |

Supplementary Table 8. Prevalence of autism with and without co-occurring intellectual disability (ID) in 8-year-old children in the United States, overall and stratified by child’s sex, Autism and Developmental Disabilities Monitoring Network, 2000-2016; multiply imputed data based on imputation of the most recent adaptive score regardless of which test or at what age the test was administered

| **Co-Occurring ID** | **Study Year** | | | | | | | | |
| --- | --- | --- | --- | --- | --- | --- | --- | --- | --- |
|  | **2000** | **2002** | **2004** | **2006** | **2008** | **2010** | **2012** | **2014** | **2016** |
| **ADDM Surveillance Definition (IQ≤70)** | | | | | | | | | |
| **Overall** | | | | | | | | | |
| ID | 3.1 (2.8-3.5) | 3.0 (2.8-3.3) | 3.5 (3.1-3.8) | 4.0 (3.7-4.3) | 5.3 (4.9-5.7) | 5.5 (5.2-5.8) | 5.2 (4.9-5.5) | 5.5 (5.2-5.8) | 6.5 (6.2-6.9) |
| No ID | 3.4 (3.0-3.7) | 3.8 (3.5-4.0) | 4.3 (4.0-4.7) | 5.5 (5.2-5.9) | 8.3 (7.8-8.7) | 11.6 (11.1-12.0) | 10.5 (10.1-10.9) | 12.0 (11.5-12.4) | 12.3 (11.9-12.8) |
| **Boys** | | | | | | | | | |
| ID | 4.4 (3.8-4.9) | 4.6 (4.2-5.0) | 5.4 (4.8-6.0) | 6.2 (5.7-6.7) | 8.3 (7.7-9.0) | 8.7 (8.2-9.3) | 8.1 (7.6-8.6) | 8.4 (7.8-8.9) | 10.1 (9.5-10.7) |
| No ID | 5.8 (5.1-6.4) | 6.4 (5.9-6.9) | 7.3 (6.6-8.0) | 9.0 (8.4-9.6) | 13.8 (13.0-14.6) | 19.0 (18.2-19.8) | 17.2 (16.5-18.0) | 19.4 (18.6-20.2) | 20.1 (19.4-20.9) |
| **Girls** | | | | | | | | | |
| ID | 1.9 (1.5-2.2) | 1.4 (1.2-1.7) | 1.5 (1.2-1.9) | 1.7 (1.5-2.0) | 2.2 (1.8-2.5) | 2.3 (2.0-2.5) | 2.2 (1.9-2.5) | 2.5 (2.2-2.8) | 2.8 (2.5-3.1) |
| No ID | 1.0 (0.7-1.2) | 1.0 (0.8-1.2) | 1.3 (1.0-1.6) | 1.8 (1.5-2.1) | 2.5 (2.1-2.8) | 3.9 (3.5-4.2) | 3.5 (3.2-3.8) | 4.3 (3.9-4.6) | 4.2 (3.8-4.6) |
| **Clinical Definition (both IQ and Adaptive Score ≤70)** | | | | | | | | | |
| **Overall** | | | | | | | | | |
| ID | 2.9 (2.6-3.2) | 2.7 (2.4-2.9) | 3.2 (2.8-3.5) | 3.4 (3.1-3.7) | 4.2 (3.9-4.6) | 4.2 (3.9-4.5) | 3.7 (3.5-4.0) | 3.9 (3.7-4.2) | 4.5 (4.2-4.8) |
| No ID | 3.6 (3.3-4.0) | 4.1 (3.9-4.4) | 4.7 (4.3-5.1) | 6.1 (5.8-6.5) | 9.4 (8.9-9.8) | 12.9 (12.5-13.4) | 12.0 (11.6-12.4) | 13.5 (13.1-14.0) | 14.3 (13.9-14.8) |
| **Boys** | | | | | | | | | |
| ID | 4.1 (3.5-4.6) | 4.0 (3.6-4.4) | 4.9 (4.3-5.4) | 5.2 (4.7-5.7) | 6.6 (6.0-7.2) | 6.6 (6.1-7.1) | 5.8 (5.4-6.2) | 5.9 (5.5-6.4) | 6.9 (6.5-7.4) |
| No ID | 6.1 (5.5-6.7) | 7.0 (6.5-7.5) | 7.8 (7.1-8.6) | 10.0 (9.3-10.7) | 15.5 (14.7-16.4) | 21.1 (20.3-22.0) | 19.5 (18.7-20.2) | 21.8 (21.0-22.6) | 23.3 (22.5-24.1) |
| **Girls** | | | | | | | | | |
| ID | 1.7 (1.4-2.1) | 1.2 (1.0-1.5) | 1.4 (1.1-1.7) | 1.5 (1.2-1.7) | 1.7 (1.4-2.0) | 1.7 (1.5-1.9) | 1.5 (1.3-1.8) | 1.9 (1.6-2.1) | 2.0 (1.8-2.3) |
| No ID | 1.1 (0.8-1.4) | 1.2 (1.0-1.4) | 1.5 (1.1-1.8) | 2.1 (1.8-2.4) | 2.9 (2.5-3.3) | 4.4 (4.0-4.8) | 4.2 (3.8-4.6) | 4.9 (4.5-5.3) | 5.0 (4.6-5.4) |

ID: intellectual disability

# Supplementary Analysis 3: Multiply imputed data limited to sites that participated in at least eight of the nine study years (Arizona, Georgia, North Carolina)

Supplementary Table 9. Prevalence of autism by adaptive level in 8-year-old children in the United States, overall and stratified by child’s sex, Autism and Developmental Disabilities Monitoring Network, 2000-2016; multiply imputed data limited to sites that participated in at least eight of the nine study years

| **Level of Adaptive Challenges** | **Study Year** | | | | | | | | |
| --- | --- | --- | --- | --- | --- | --- | --- | --- | --- |
|  | **2000** | **2002** | **2004** | **2006** | **2008** | **2010** | **2012** | **2014** | **2016** |
| **Overall** | | | | | | | | | |
| None | 0.7 (0.5-0.9) | 0.6 (0.4-0.7) | 0.8 (0.6-1.0) | 1.4 (1.2-1.7) | 3.0 (2.7-3.3) | 3.4 (3.1-3.8) | 3.6 (3.2-4.0) | 3.9 (3.5-4.3) | 4.0 (3.5-4.6) |
| Borderline | 1.5 (1.2-1.8) | 1.4 (1.1-1.6) | 2.2 (1.8-2.5) | 3.2 (2.8-3.6) | 4.8 (4.3-5.2) | 6.1 (5.6-6.5) | 6.3 (5.8-6.8) | 6.0 (5.4-6.5) | 7.8 (7.1-8.6) |
| Mild | 3.0 (2.6-3.4) | 3.6 (3.2-4.0) | 4.6 (4.1-5.1) | 4.9 (4.5-5.4) | 4.9 (4.5-5.3) | 5.6 (5.1-6.0) | 5.2 (4.7-5.6) | 5.7 (5.2-6.2) | 7.2 (6.5-7.9) |
| Moderate to Profound | 1.4 (1.1-1.6) | 1.3 (1.1-1.6) | 1.6 (1.3-1.9) | 1.4 (1.2-1.7) | 1.0 (0.9-1.2) | 1.1 (0.9-1.3) | 0.8 (0.6-1.0) | 0.9 (0.7-1.1) | 1.2 (0.9-1.5) |
| **Boys** | | | | | | | | | |
| None | 1.2 (0.9-1.6) | 1.0 (0.7-1.3) | 1.3 (0.9-1.7) | 2.4 (1.9-2.8) | 4.9 (4.4-5.5) | 5.8 (5.1-6.4) | 5.9 (5.2-6.5) | 6.3 (5.6-7.0) | 6.8 (5.8-7.8) |
| Borderline | 2.5 (2.0-3.1) | 2.3 (1.9-2.8) | 3.7 (3.1-4.4) | 5.2 (4.6-5.9) | 7.9 (7.2-8.7) | 10.1 (9.2-10.9) | 10.4 (9.5-11.2) | 9.8 (8.9-10.7) | 12.7 (11.3-14) |
| Mild | 4.8 (4.1-5.5) | 5.9 (5.2-6.5) | 7.3 (6.4-8.1) | 7.8 (7.1-8.6) | 7.9 (7.1-8.6) | 8.9 (8.1-9.7) | 8.4 (7.6-9.1) | 9.0 (8.1-9.8) | 11.3 (10.1-12.6) |
| Moderate to Profound | 1.9 (1.5-2.3) | 2.0 (1.7-2.4) | 2.4 (1.9-2.9) | 2.3 (1.9-2.7) | 1.6 (1.2-1.9) | 1.7 (1.4-2.0) | 1.3 (1.0-1.6) | 1.4 (1.0-1.7) | 1.9 (1.4-2.4) |
| **Girls** | | | | | | | | | |
| None | 0.2 (0.1-0.4) | 0.2 (0.0-0.3) | 0.3 (0.1-0.4) | 0.5 (0.3-0.8) | 1.0 (0.7-1.2) | 1.1 (0.8-1.4) | 1.3 (1.0-1.6) | 1.5 (1.2-1.9) | 1.2 (0.8-1.7) |
| Borderline | 0.5 (0.3-0.8) | 0.4 (0.2-0.6) | 0.6 (0.3-0.8) | 1.1 (0.8-1.4) | 1.5 (1.1-1.8) | 2.0 (1.6-2.4) | 2.2 (1.8-2.6) | 2.0 (1.6-2.4) | 2.9 (2.2-3.5) |
| Mild | 1.2 (0.8-1.5) | 1.3 (1.0-1.6) | 1.9 (1.5-2.3) | 1.9 (1.5-2.3) | 1.8 (1.5-2.2) | 2.2 (1.8-2.6) | 1.9 (1.5-2.3) | 2.3 (1.8-2.7) | 3.0 (2.3-3.7) |
| Moderate to Profound | 0.9 (0.6-1.2) | 0.7 (0.4-0.9) | 0.7 (0.5-1.0) | 0.6 (0.4-0.8) | 0.5 (0.3-0.7) | 0.5 (0.3-0.7) | 0.3 (0.2-0.5) | 0.4 (0.3-0.6) | 0.5 (0.2-0.8) |

Supplementary Table 10. Prevalence of autism with and without co-occurring intellectual disability (ID) in 8-year-old children in the United States, overall and stratified by child’s sex, Autism and Developmental Disabilities Monitoring Network, 2000-2016; multiply imputed data limited to sites that participated in at least eight of the nine study years

| **Co-Occurring ID** | **Study Year** | | | | | | | | |
| --- | --- | --- | --- | --- | --- | --- | --- | --- | --- |
|  | **2000** | **2002** | **2004** | **2006** | **2008** | **2010** | **2012** | **2014** | **2016** |
| **ADDM Surveillance Definition (IQ≤70)** | | | | | | | | | |
| **Overall** | | | | | | | | | |
| ID | 2.9 (2.5-3.3) | 3.1 (2.8-3.4) | 3.9 (3.4-4.3) | 4.3 (3.9-4.7) | 5.2 (4.8-5.6) | 5.5 (5.1-6.0) | 5.4 (5.0-5.8) | 5.4 (5.0-5.9) | 7.2 (6.5-7.9) |
| No ID | 3.7 (3.3-4.1) | 3.7 (3.4-4.1) | 5.2 (4.7-5.7) | 6.6 (6.2-7.1) | 8.5 (7.9-9.0) | 10.6 (10.0-11.2) | 10.4 (9.9-11.0) | 11.0 (10.4-11.6) | 12.9 (12.0-13.9) |
| **Boys** | | | | | | | | | |
| ID | 4.1 (3.5-4.7) | 4.9 (4.3-5.5) | 6.0 (5.2-6.8) | 6.7 (6.0-7.4) | 8.1 (7.4-8.9) | 8.7 (7.9-9.4) | 8.5 (7.8-9.3) | 8.4 (7.6-9.2) | 11.2 (10.0-12.4) |
| No ID | 6.3 (5.6-7.1) | 6.3 (5.6-6.9) | 8.6 (7.7-9.6) | 10.9 (10.0-11.7) | 14.1 (13.1-15.0) | 17.7 (16.7-18.8) | 17.3 (16.3-18.4) | 18.0 (16.9-19.1) | 21.3 (19.7-22.9) |
| **Girls** | | | | | | | | | |
| ID | 1.7 (1.4-2.1) | 1.3 (1.0-1.6) | 1.7 (1.3-2.1) | 1.9 (1.5-2.2) | 2.1 (1.7-2.5) | 2.3 (1.9-2.7) | 2.3 (1.9-2.7) | 2.4 (2.0-2.8) | 3.1 (2.4-3.8) |
| No ID | 1.0 (0.7-1.2) | 1.1 (0.8-1.4) | 1.6 (1.2-2.0) | 2.2 (1.8-2.6) | 2.6 (2.2-3.0) | 3.4 (2.9-3.9) | 3.4 (2.9-3.9) | 3.8 (3.2-4.3) | 4.3 (3.6-5.1) |
| **Clinical Definition (both IQ and VABS 6-8yrs Composite ≤70)** | | | | | | | | | |
| **Overall** | | | | | | | | | |
| ID | 2.7 (2.4-3.0) | 2.9 (2.5-3.2) | 3.4 (3.0-3.8) | 3.6 (3.3-4.0) | 3.8 (3.4-4.2) | 4.0 (3.7-4.4) | 3.5 (3.1-3.9) | 3.9 (3.5-4.3) | 5.0 (4.4-5.6) |
| No ID | 3.9 (3.5-4.3) | 4.0 (3.6-4.4) | 5.6 (5.1-6.1) | 7.3 (6.8-7.8) | 9.8 (9.3-10.4) | 12.1 (11.5-12.8) | 12.3 (11.7-13.0) | 12.5 (11.8-13.2) | 15.2 (14.2-16.1) |
| **Boys** | | | | | | | | | |
| ID | 3.8 (3.3-4.4) | 4.5 (3.9-5.0) | 5.3 (4.5-6.0) | 5.7 (5.1-6.3) | 6.0 (5.3-6.6) | 6.3 (5.7-7.0) | 5.5 (4.9-6.1) | 6.0 (5.3-6.7) | 7.8 (6.7-8.8) |
| No ID | 6.6 (5.8-7.3) | 6.7 (6.0-7.4) | 9.4 (8.4-10.3) | 11.9 (11.0-12.8) | 16.3 (15.3-17.3) | 20.1 (18.9-21.2) | 20.4 (19.2-21.5) | 20.4 (19.2-21.6) | 24.8 (23.0-26.5) |
| **Girls** | | | | | | | | | |
| ID | 1.6 (1.2-1.9) | 1.2 (0.9-1.5) | 1.6 (1.2-1.9) | 1.5 (1.2-1.8) | 1.6 (1.3-1.9) | 1.7 (1.4-2.0) | 1.5 (1.2-1.8) | 1.8 (1.4-2.1) | 2.2 (1.6-2.8) |
| No ID | 1.1 (0.8-1.4) | 1.2 (0.9-1.5) | 1.8 (1.4-2.2) | 2.5 (2.1-3.0) | 3.1 (2.7-3.6) | 4.0 (3.5-4.5) | 4.1 (3.6-4.7) | 4.4 (3.8-5.0) | 5.2 (4.4-6.1) |

ID: intellectual disability
